# Phosphorylation of Mitochondrial Polyubiquitin by PINK1 Promotes Parkin Mitochondrial Tethering

**DOI:** 10.1371/journal.pgen.1004861

**Published:** 2014-12-04

**Authors:** Kahori Shiba-Fukushima, Taku Arano, Gen Matsumoto, Tsuyoshi Inoshita, Shigeharu Yoshida, Yasushi Ishihama, Kwon-Yul Ryu, Nobuyuki Nukina, Nobutaka Hattori, Yuzuru Imai

**Affiliations:** 1 Department of Neurology, Juntendo University Graduate School of Medicine, Tokyo, Japan; 2 Department of Research for Parkinson's Disease, Juntendo University Graduate School of Medicine, Tokyo, Japan; 3 Department of Neuroscience for Neurodegenerative Disorders, Juntendo University Graduate School of Medicine, Tokyo, Japan; 4 Department of Molecular and Cellular BioAnalysis, Graduate School of Pharmaceutical Sciences, Kyoto University, Kyoto, Japan; 5 Department of Life Science, University of Seoul, Seoul, Korea; Stanford University School of Medicine, United States of America

## Abstract

The kinase PINK1 and the E3 ubiquitin (Ub) ligase Parkin participate in mitochondrial quality control. The phosphorylation of Ser65 in Parkin's ubiquitin-like (UBl) domain by PINK1 stimulates Parkin activation and translocation to damaged mitochondria, which induces mitophagy generating polyUb chain. However, Parkin Ser65 phosphorylation is insufficient for Parkin mitochondrial translocation. Here we report that Ser65 in polyUb chain is also phosphorylated by PINK1, and that phosphorylated polyUb chain on mitochondria tethers Parkin at mitochondria. The expression of Tom70^MTS^-4xUb SE, which mimics phospho-Ser65 polyUb chains on the mitochondria, activated Parkin E3 activity and its mitochondrial translocation. An E3-dead form of Parkin translocated to mitochondria with reduced membrane potential in the presence of Tom70^MTS^-4xUb SE, whereas non-phospho-polyUb mutant Tom70^MTS^-4xUb SA abrogated Parkin translocation. Parkin binds to the phospho-polyUb chain through its RING1-In-Between-RING (IBR) domains, but its RING0-linker is also required for mitochondrial translocation. Moreover, the expression of Tom70^MTS^-4xUb SE improved mitochondrial degeneration in *PINK1*-deficient, but not *Parkin*-deficient, *Drosophila*. Our study suggests that the phosphorylation of mitochondrial polyUb by PINK1 is implicated in both Parkin activation and mitochondrial translocation, predicting a chain reaction mechanism of mitochondrial phospho-polyUb production by which rapid translocation of Parkin is achieved.

## Author Summary

Parkinson's disease is a neurodegenerative disorder caused by degeneration of the midbrain dopaminergic system in addition to other nervous systems. *PINK1* and *parkin*, which encode mitochondrial protein kinase and cytosolic Ub ligase, respectively, were identified as the genes responsible for the autosomal recessive form of juvenile Parkinson's disease. Activation of PINK1 upon reduction of mitochondrial membrane potential recruits Parkin from the cytosol activating its Ub ligase activity, which ensures removal of damaged mitochondria through mitophagy. However, how PINK1 recruits Parkin to the damaged mitochondria remained unclear. Here, we describe that the phosphorylation of polyUb chain by PINK1 is a key event to recruit Parkin on the mitochondria. Parkin binds to, and is activated by, phospho-polyUb generated by Parkin in collaboration with PINK1. Expression of a phospho-polyUb mimetic protein on mitochondria rescued mitochondrial degeneration caused by loss of PINK1 in *Drosophila*. Our study suggests the existence of an amplification cascade of Parkin activation and mitochondrial translocation, in which a ‘seed' of phosphorylated polyUb on the mitochondria, generated by PINK1 and Parkin, triggers a chain reaction of Parkin recruitment and activation.

## Introduction

Parkin (Gene ID: 5071) is an RBR (RING-in-between-RING) E3 Ub ligase with a Ubl domain at its N-terminus, and an atypical RING domain, RING0, has been newly identified in the linker region between the Ubl and the RBR domains [1], [2]. Mutations in *parkin* genes cause early-onset Parkinson's disease (PD) [3]. *Drosophila* genetics and cell biological studies have revealed that Parkin regulates mitochondrial homeostasis in collaboration with another early onset-PD gene product, PINK1 (Gene ID: 65018) [4]–[11]. PINK1, which is a serine/threonine protein kinase with a mitochondrial target sequence [12], is constitutively processed by the mitochondrial proteases at the mitochondrial membrane of healthy mitochondria, resulting in proteasomal degradation [10], [13], [14]. The reduction in mitochondrial membrane potential (ΔΨm) in damaged mitochondria leads to the accumulation and activation of PINK1 on the mitochondrial outer membrane [10], [15], [16]. The activated PINK1 recruits Parkin from the cytosol to the mitochondria upon decreased membrane potential, which stimulates Parkin E3 activity, promoting mitochondrial degradation via an autophagic event known as mitophagy [7]–[11]. The recruitment of cytosolic Parkin to the mitochondria upon disruption of ΔΨm is thought to be the first step of mitophagy for the removal of damaged mitochondria. Translocated Parkin leads to polyUb accumulation on the mitochondria [9], which further recruits Ub-proteasome- and autophagy-related proteins for mitochondrial elimination, including the 26S proteasome, p97/VCP, p62/SQSTM1, LC3, ATG5 and ATG7 [8]–[10], [17]–[20].

The RBR-containing E3 family proteins, including Parkin, have recently been proposed to possess a hybrid E3 activity with the properties of both HECT-type and RING-finger type E3s [21], [22]. Similar to HECT-type E3s, Parkin can form a catalytic intermediate thioester between the Cys431 residue and the C-terminus of Ub, whereby Ub appears to be directly transferred to the substrate. The Ubl domain has been shown to autoinhibit the C-terminal RBR-containing region intramolecularly [23]. We and others have reported that PINK1 directly phosphorylates Parkin at Ser65 in the Ubl domain [15], [24], whereby the autoinhibition mechanism of the Ubl domain appears to be weakened. However, another factor(s) regulated by PINK1 is expected to execute Parkin mitochondrial translocation because the mitochondrial translocation of phosphomimetic Parkin and Parkin lacking the Ubl domain still requires PINK1 activation [24].

To address this issue, we searched for PINK1 kinase substrates other than Parkin as possible regulators of the mitochondrial translocation of Parkin. Herein, we report that Ser65 of Ub is phosphorylated by PINK1, which has also been identified by the other groups [25]–[27]. Moreover, we show that phospho-polyUb chain formation on the mitochondria activates Parkin E3 ligase and facilitates mitochondrial translocation of Parkin. Furthermore, mitochondrial expression of the phospho-mimetic polyUb chain ameliorated the mitochondrial degeneration caused by the loss of PINK1 in *Drosophila*, suggesting that endogenous Parkin is activated by and recruited to the phospho-polyUb chain. Our study proposes an amplification cascade of Parkin activation and mitochondrial translocation, in which a ‘seed’ of phosphorylated polyUb on the mitochondria, generated by PINK1 and Parkin, triggers a chain reaction of Parkin recruitment and activation.

## Results

### Ub is phosphorylated by PINK1 upon depolarization in ΔΨm

**Figure 1:**
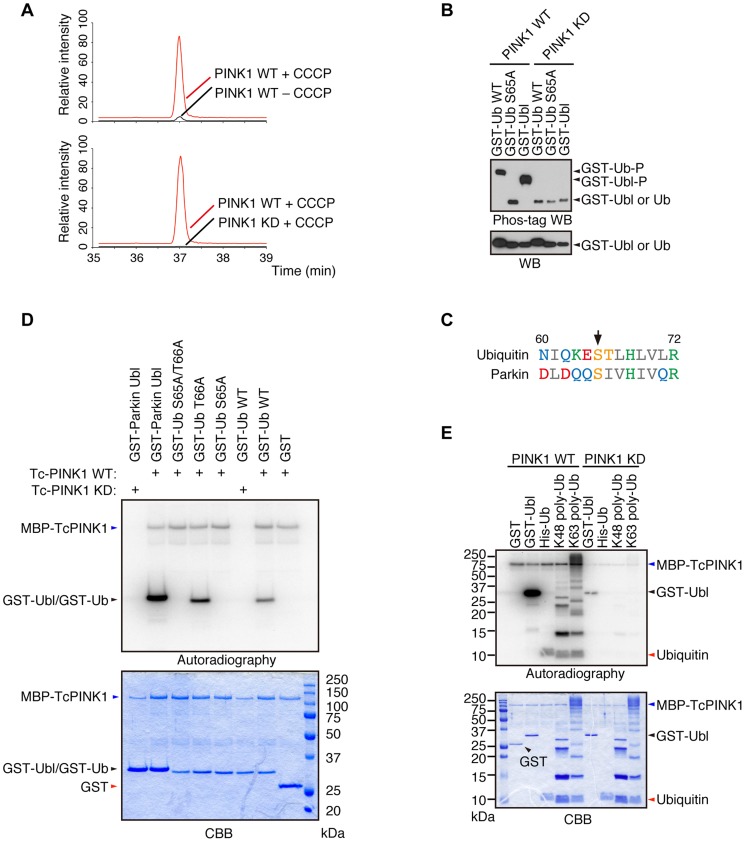
Ub is a substrate of PINK1. (**A**) Mass spectrometric screening of PINK1 substrates identified Ub phosphorylation at Ser65. Extracted ion chromatograms of dimethylated EpSTLHLVLR from mouse Ub phosphorylated by WT or KD PINK1 with or without CCCP treatment. Ub phosphorylation was specifically increased in PINK1-FLAG WT/*PINK1^−/−^*MEFs treated with CCCP. The peak area ratios of PINK1 WT+ CCCP to PINK1 WT – CCCP and PINK1 WT+ CCCP to PINK1 KD+ CCCP are 22.3 and 308.8, respectively. (**B**) Recombinant glutathione S-transferase (GST), GST-fusion Ub (Ub, WT and S65A) and GST-fusion Parkin Ubl domains were incubated with TcPINK1 WT or KD in 40 µl of kinase reaction buffer (50 mM Tris-HCl, pH 7.5, 0.1 mM EGTA, 10 mM MgCl_2_, 2 mM DTT and 2 mM ATP) for 90 min at 30°C, and they were subsequently separated on a Phos-tag gel and a conventional polyacrylamide gel, followed by western blotting with anti-GST antibodies. (**C**) Alignment of the amino acid sequences surrounding Ser65 (marked by an arrow) from human Ub and human Parkin. The numbers correspond to the residue numbers in the Parkin and Ub proteins. Acidic, basic and hydrophobic amino acids are shown in red, green and grey, respectively. Amino acids with amido and hydroxyl groups are shown in blue and yellow, respectively. (**D**) Ser65 of Ub is the only site phosphorylated by PINK1. The GST, GST-Ub (WT, S65A, T66A and S65A/T66A) and Parkin Ubl domains were incubated with TcPINK1 WT or KD as in (**A**). The ATP source used was 10 µCi of γ-^32^P ATP. Phosphorylated Ub was detected by autoradiography (^32^P). The reaction mixtures were subjected to SDS-PAGE, CBB staining and autoradiography. (**E**) Both K48- and K63-linked polyUb chains were phosphorylated by PINK1. The kinase assay was performed as in (**D**). His-Ub; recombinant 6× His-tagged Ub.

We revealed that Parkin is phosphorylated at Ser65 in the Ubl domain by PINK1 [24]. Although the replacement of Parkin Ser65 with alanine to block the phosphorylation effect impaired the mitochondrial translocation of Parkin in cultured mammalian cells, Parkin translocation and subsequent mitophagy eventually occur, suggesting that there is another factor(s) regulated by PINK1. We searched for PINK1 substrates that could be regulators of Parkin in mouse embryonic fibroblasts (MEFs) derived from *PINK1*-deficient mice, in which FLAG-tagged wild-type (WT) or kinase-dead (KD; triple mutant with K219A, D362A and D384A) forms of PINK1 were virally introduced (hereafter referred to as “PINK1-FLAG WT/*PINK1^−/−^*” and “PINK1-FLAG KD/*PINK1^−/−^*” MEFs, respectively) followed by treatment with the protonophore carbonyl cyanide *m*-chlorophenyl hydrazone (CCCP) to activate PINK1 or with DMSO as a control. The cell pellets were then subjected to mass spectrometric analysis to discover phospho-peptides specific to the PINK1 activation condition. As shown in Fig. 1A, Ub was specifically phosphorylated at Ser65 in CCCP-treated PINK1-FLAG WT/*PINK1^−/−^* MEFs but not in PINK1-FLAG KD/*PINK1^−/−^* MEFs. Similar results were obtained in a comparison of CCCP-treated and DMSO-treated PINK1-FLAG WT/*PINK1^−/−^* MEFs. Phos-tag Western blotting, in which phosphorylated proteins appear as slower migrating bands [24], revealed that Ub at Ser65 and Parkin at Ser65 in the Ubl domain were directly phosphorylated by PINK1 (Fig. 1B). Although Ub Ser65 corresponds to Parkin Ubl Ser65 in an alignment by a computational homology program, there are several divergences in the flanking sequence between Ub and the Parkin Ubl domain (Fig. 1C). The unique Thr66 in Ub is a potential phosphorylation site, and the mass spectrometric analysis could not exclude the possibility of Thr66 phosphorylation. However, the replacement of Thr66 in Ub with alanine was not affected by PINK1 phosphorylation, indicating that Ub Ser65 is a major phosphorylation site for PINK1 (Fig. 1D) [15]. The accumulation of Lys(K)63-linked and K48-linked polyUb chains on the mitochondria is a prominent phenomenon during mitophagy. We examined whether these Ub chains are also phosphorylated by PINK1 (Fig. 1E) [20]. Because the K63-linked isopeptide bond is close to Ser65, the phosphorylation efficiency may be different between K63- and K48-polyUb. However, PINK1 could phosphorylate both polyUb chains similarly based on the ratio of intensity of autoradiography to density of CBB staining (Fig. 1E).

### Phosphorylation of Ser65 in Ub and Parkin additively activates Parkin E3

After establishing that Ub and Parkin are phosphorylated at Ser65 by PINK1, we examined whether the phosphorylation of both Parkin and Ub is required for the Parkin translocation to mitochondria. The activation of Parkin E3 activity can be assessed as the autoubiquitination of N-terminal GFP-tagged Parkin, in which the GFP moiety acts as a pseudo-substrate for Parkin [9], [18]. The Ser65 residues of GFP-Parkin and HA-Ub were replaced with alanine (SA) or glutamate (SE) to create non-phosphorylated or phosphomimetic forms, respectively, of the two proteins. The expression of the three types (WT, SA and SE) of GFP-Parkin alone did not show autoubiquitination signals (S1A Figure, lanes 1, 5 and 9). The autoubiquitination of GFP-Parkin WT and SA was observed with the coexpression of the three types (WT, SA and SE) of HA-Ub (S1A Figure, lanes 2–4, 6–8). The autoubiquitination by HA-Ub coexpression was facilitated in GFP-Parkin SE (S1A Figure, lanes 10–12 *vs.* lanes 2–4, 6–8), and this result was most prominent for the combination of GFP-Parkin SE and HA-Ub SE (S1A Figure, lane 12). Under this experimental condition, the ubiquitination and degradation of the known Parkin substrate Mitofusin1 (Mfn1) were not observed, suggesting that Parkin is activated in the cytosol without mitochondrial translocation. During the preparation of this manuscript, Kazlauskaite, *et al.,* Kane *et al.* and Koyano et al, reported that monoUb phosphorylated by PINK1 is sufficient for Parkin E3 activation in reconstitution assays. Our results are also consistent with them [25]–[27]. Parkin E3 activity is required for the mitochondrial translocation of Parkin because the E3-deficient forms of Parkin, C431F or C431S, exhibit no translocation activity [22]. However, even the coexpression of Parkin SE and Ub SE did not stimulate the mitochondrial translocation of Parkin, indicating that at least one other factor is required for its mitochondrial translocation (S1B Figure).

### PolyUb phosphorylated at Ser65 is required for the mitochondrial translocation of Parkin

We next examined whether Ub is required for Parkin translocation. The transient inhibition of Ub expression by siRNA significantly inhibited Parkin translocation after CCCP treatment (S2A Figure). Because the longer inhibition of Ub expression results in cell death, we also used MEFs lacking the stress-inducible polyUb gene *Ubc*, in which steady-state Ub levels were reduced by 40% compared with WT cells [28]. The mitochondrial translocation of GFP-Parkin was significantly delayed in *Ubc^−/−^* MEFs compared with *Ubc^+/+^* MEFs upon CCCP treatment (S2B,C Figure). These results suggested that Ub is required for the mitochondrial translocation of Parkin.

**Figure 2:**
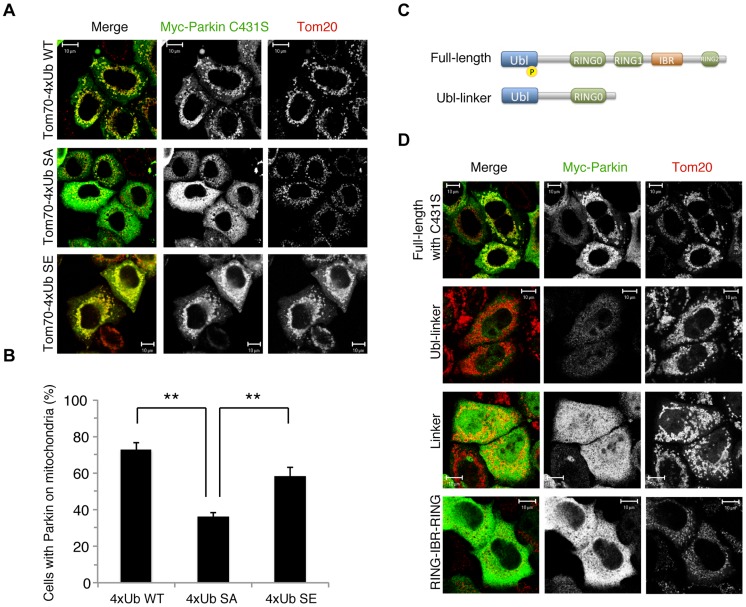
Phospho-polyUb chain mimic stimulates the mitochondrial translocation of Parkin independently of Parkin E3 activity. (**A**) The mitochondrial expression of the non-phosphorylated form of polyUb inhibits Parkin translocation. HeLa cells transfected with Myc-Parkin C431S along with Tom70^MTS^-4xUb WT, SA or SE were treated with 10 µM valinomycin for 3 hr. (**B**) Quantification of the mitochondrial translocation efficiency of Parkin C431S. The graph shows the means ±SEM of the percentages of cells exhibiting mitochondrial recruitment in three independent experiments, with ∼100 anti-Myc staining-positive cells counted per sample. ** *p*<0.01. (**C**) Diagram of the Parkin protein illustrating the pathogenic mutants used in (**D**). (**D**) Full-length Parkin is required for mitochondrial translocation. HeLa cells transfected with the indicated mutant forms of Parkin along with Tom70^MTS^-4xUb WT were treated with 10 µM valinomycin for 3 hr. Scale bars  = 10 µm in (**A, D**).

The results of a recent study suggest the formation of K63-linked polyUb chains on mitochondria is involved in Parkin mitochondrial translocation [29]. The mitochondrial expression of quarterly repeated Ub chain, which mimics K63-linked polyUb, recruits Parkin in a PINK1-activity-dependent manner, under which condition Parkin E3 activity is not required [29]. Four tandem copies of Ub G76V with the mitochondrial targeting sequence of Tom70 and 2 copies of FLAG-tag (Tom70^MTS^-4xUb WT) along with Myc-tagged Parkin C431S were co-transfected into HeLa cells, which do not express endogenous Parkin. As reported, Parkin C431S was recruited to the mitochondria when the cells were treated with 10 µM CCCP or valinomycin (Fig. 2A,B) [29]. Intriguingly, the replacement of Ub Ser65 with alanine (Tom70^MTS^-4xUb SA) but not glutamate (Tom70^MTS^-4xUb SE) significantly impaired Parkin translocation (Fig. 2A,B). The mitochondrial expression of monoUb or the cytosolic expression of 4xUb did not lead to efficient Parkin translocation (S3A,C,D Figure) and the Parkin translocation was not observed in PINK1^−/−^ cells expressing Tom70^MTS^-4xUb WT (S3B Figure) or Tom70^MTS^-4xUb SE (S3C,D Figure). These results suggest that PINK1-mediated phosphorylation of both Parkin and polyUb chain is required for Parkin mitochondrial translocation.

**Figure 3:**
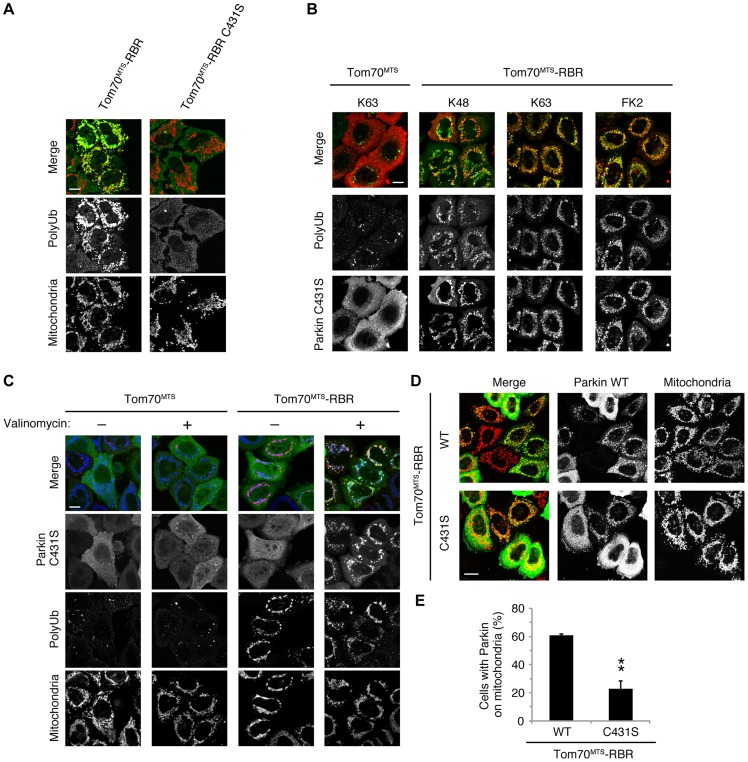
Phospho-polyUb chain on the mitochondria induces the mitochondrial translocation of Parkin. (**A**) Mitochondrial targeting of the Parkin RBR domain generates polyUb chain at the mitochondria in an E3 activity-dependent manner. Mitochondrial Ub chains (green) and mitochondria (red) were visualized with anti-polyUb (FK2) and anti-Tom20, respectively, in HeLa cells transfected with Tom70^MTS^-RBR or Tom70^MTS^-RBR C431S. The polyUb and Tom20 (mitochondria) signals are also shown as monochrome images. (**B**) Tom70^MTS^-RBR synthesizes both K48 and K63 Ub linkages. HeLa cells transfected with Tom70^MTS^ or Tom70^MTS^-RBR along with 3xMyc-Parkin C431S were treated as in Fig. 2. Mitochondrial Ub chains (green) were visualized with anti-polyUb (FK2), anti-K48-linkeage specific (K48) and anti-K63-linkeage specific (K63) polyUb. Parkin C431S was visualized with anti-Myc (red). (**C**) HeLa cells transfected with Tom70^MTS^ or Tom70^MTS^-RBR along with 3xMyc-Parkin C431S were treated with or without 10 µM valinomycin for 2 hr. 3xMyc-Parkin C431S, polyUb and the mitochondria were visualized with anti-Myc (green), anti-polyUb (red), and anti-Tom20 (blue), respectively. (**D, E**) Mitochondrial phospho-polyUb generated by Tom70^MTS^-RBR accelerates Parkin translocation. HeLa cells transfected with 3xMyc-Parkin WT along with E3 active Tom70^MTS^-RBR WT or E3-dead Tom70^MTS^-RBR C431S were treated with 10 µM valinomycin for 40 min. (**D**) Parkin (green) and mitochondria (red) were visualized with anti-Myc and anti-Tom20, respectively. Each signal is also shown as monochrome images. (**E**) Quantification of the mitochondrial translocation efficiency of Parkin as in (**D**). The graph shows the means ±SEM of the percentages of cells exhibiting mitochondrial recruitment in three independent experiments, with ∼100 anti-Myc staining-positive cells counted per sample. ** *p*<0.01 (two-tailed unpaired Student's *t*-test). Scale bars  = 10 µm in (**A–D**).

**Figure 4:**
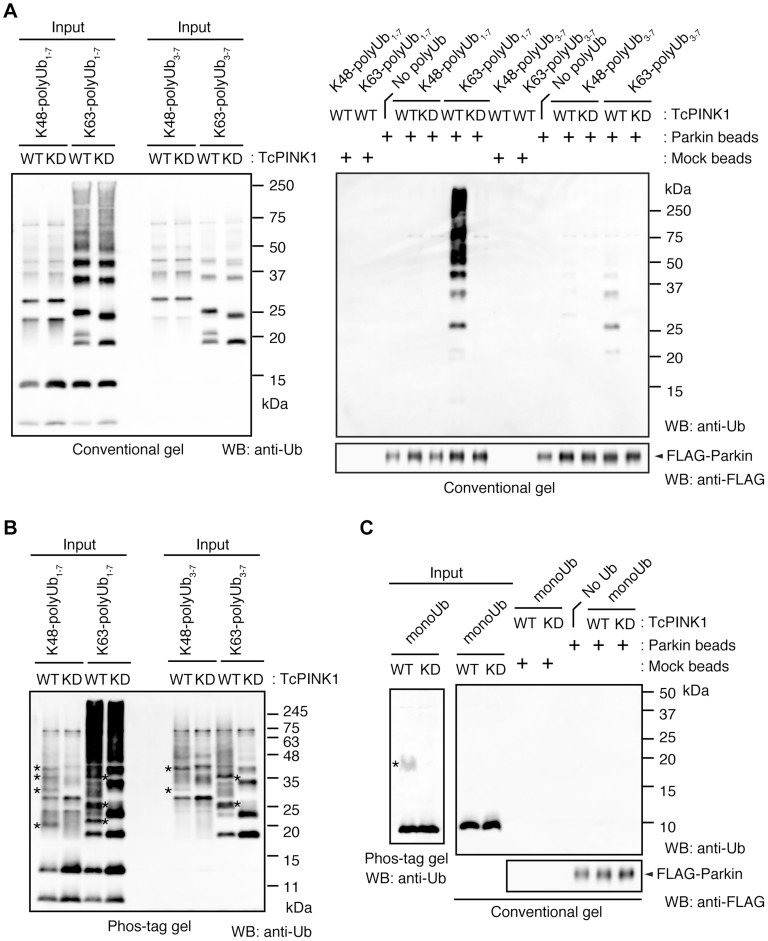
Parkin preferentially binds to K63-linked polyUb phosphorylated by PINK1. (**A**) *In vitro* Parkin pull-down assay for linkage-specific phospho-Ub chains. Linkage-specific Ub chains (K48- and K63-linked Ub_1-7_, and K48- and K63-linked Ub_3-7_) treated with TcPINK1 WT or KD were pull-down with FLAG-Parkin-conjugated beads (Parkin beads) or mock beads. A control pull-down of FLAG-Parkin without polyUb chains was also included (No polyUb). Note that the commercial product K63-linked Ub_1-7_ contains longer Ub chains than K48-linked Ub_1-7_ (left). (**B**) Phosphorylation of Ub chains (asterisks) was confirmed by Phos-tag western blot. (**C**) *In vitro* Parkin pull-down assay for monoUb. Bovine Ub treated with TcPINK1 WT or KD was pull-down with FLAG-Parkin as in (**A**). Phosphorylation of monoUb (asterisk) was confirmed by Phos-tag western blot (left).

**Figure 5:**
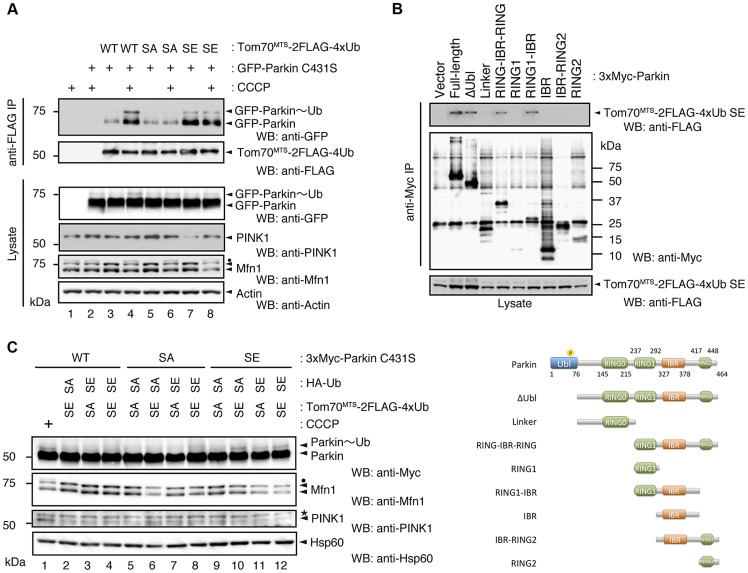
Phosphorylation of the polyUb chain activates Parkin E3 activity. (**A**) The Ub SA mutation attenuates the CCCP-dependent Parkin-polyUb association, whereas the Ub SE mutation promotes this association. HEK293T cells were transfected with the indicated combination of plasmids and were then treated with 10 µM CCCP for 1 hr. Tom70^MTS^-2FLAG-4Ub constructs (WT, SA and SE) were immunoprecipitated with anti-FLAG beads and eluted with 100 µg/ml 3xFLAG peptide. Coprecipitated GFP-Parkin was detected by western blot. Note that the expression of Tom70^MTS^-2FLAG-4Ub SE alone did not lead to PINK1 accumulation (lane 7). The dot indicates putative ubiquitinated Mfn1. (**B**) Parkin associates with Tom70^MTS^-2FLAG-4Ub SE through its RING1-IBR. HEK293T cells were transfected with a series of 3xMyc-tagged full-length Parkin and its mutants together with Tom70^MTS^-2FLAG-4Ub SE. Coprecipitated Tom70^MTS^-2FLAG-4Ub SE was detected with an anti-FLAG antibody. Diagram of the Parkin deletion mutants used in this experiment is shown at the bottom. (**C**) Phosphomimetic Parkin SE is activated by either cytosolic SE Ub or the mitochondrial SE polyUb chain. Note that the accumulation of PINK1 and obvious degradation of Mfn1 were not observed, even with a combination of HA-Ub SE and Tom70^MTS^-2FLAG-4xUb SE (lane 12 *vs.* lane 1). The dot and the asterisk indicate putative ubiquitinated Mfn1 and non-specific signals, respectively.

We next identified which domain of Parkin is responsible for binding to the phospho-polyUb chain using artificial truncated mutants (Fig. 2C,D, Ubl-linker, Linker and RING-IBR-RING, see also Fig. 5B). Tom70^MTS^-4xUb WT was co-expressed with Parkin truncated mutants in HeLa cells, and the cells were then treated with valinomycin to achieve the PINK1-mediated phosphorylation of Tom70^MTS^-4xUb WT. We found that none of the truncated mutants showed mitochondrial translocation upon the generation of phospho-polyUb on the mitochondria (Fig. 2D). We further confirmed whether the mitochondrial expression of native polyUb chains phosphorylated by PINK1 leads to Parkin C431S recruitment (Fig. 3). Parkin RING-IBR-RING has a constitutive E3 activity [30], [31]. Parkin RING-IBR-RING fused with the mitochondrial targeting sequence of Tom70 (Tom70^MTS^-RBR) or Tom70^MTS^ alone together with GFP-Parkin C431S were expressed in HeLa cells. The expression of the E3 activity of Tom70^MTS^-RBR was accompanied by a prominent accumulation of K63-linked and K48-linked polyUb chains on the mitochondria (Fig. 3A,B). The cells were then treated with valinomycin to achieve the PINK1-mediated phosphorylation as in Fig. 2A, by which Parkin C431S was translocated to the mitochondria with polyUb accumulation only when PINK1 is activated, supporting the idea that phosphorylated polyUb chain is required for Parkin translocation (Fig. 3C). A previous report showed that Parkin lacking its E3 activity binds to the RING-IBR-RING domain of Parkin on the mitochondria [22]. Given that Parkin moves to mitochondria by direct binding to the mitochondrial Parkin RING-IBR-RING, Parkin would translocate to the mitochondria expressing an E3-dead form of Tom70^MTS^-RBR (Tom70^MTS^-RBR C431S) with the same efficiency as Tom70^MTS^-RBR. However, Parkin translocation was accelerated by Tom70^MTS^-RBR compared with Tom70^MTS^-RBR C431S upon valinomycin treatment, suggesting that Parkin moves to mitochondria recognizing polyUb phosphorylated by PINK1 rather than the mitochondrial Parkin RING-IBR-RING (Fig. 3D,E). Because PINK1 could phosphorylate both K63-linked and K48-linked polyUb chains *in vitro* (Fig. 1E), we next examined whether Parkin binds to linkage-specific polyUb chains. Recombinant monoUb and K63-linked and K48-linked polyUb chains incubated with recombinant PINK1 WT or KD were added to a solution containing FLAG-Parkin-conjugated agarose beads and a FLAG-Parkin pull-down assay was performed. The results revealed that FLAG-Parkin preferentially binds to K63-linked polyUb chains, especially to longer chains (Fig. 4A,B), whereas FLAG-Parkin failed to interact with phospho-monoUb (Fig. 4C). These results suggest that K63-linked polyUb phosphorylated by PINK1 is a major mitochondrial anchoring factor for Parkin. However, Parkin purified from bacteria, but not FLAG-Parkin from cultured cells, exhibited a similar affinity for both phospho-chains (S4 Figure). The possible reasons will be discussed in Discussion section.

In a similar experimental setting to the cytochemical analysis with HeLa cells in Fig. 2A, the association of GFP-Parkin C431S with mitochondrially targeted 4xUb (Tom70^MTS^-2FLAG-4Ub WT, SA or SE) was determined with and without CCCP treatment in HEK293T cells, which weakly express endogenous Parkin. Although GFP-Parkin C431S alone was not precipitated by anti-FLAG-conjugated agarose beads (Fig. 5A, lane 2), a weak association of GFP-Parkin C431S with Tom70^MTS^-2FLAG-4Ub WT was observed (Fig. 5A, lane 3), and CCCP treatment enhanced this association (Fig. 5A, lane 4). The association of GFP-Parkin C431S with Tom70^MTS^-2FLAG-4Ub SA was comparable with that with Tom70^MTS^-2FLAG-4Ub WT (Fig. 5A, lane 5 *vs.* lane 3). However, enhancement of the association between GFP-Parkin C431S and Tom70^MTS^-2FLAG-4Ub SA was not observed upon CCCP treatment (Fig. 5A, lane 6 *vs.* lane 5). In contrast, a strong association of GFP-Parkin C431S with Tom70^MTS^-2FLAG-4Ub SE was observed even under steady-state conditions and was unchanged by CCCP treatment (Fig. 5A, lane 7 *vs.* lane 8). Under these conditions, the CCCP-independent charge of Ub to GFP-Parkin C431S was observed, suggesting that endogenous Parkin E3 activity could also be activated in this setting (Fig. 5A, lane 7, GFP-Parkin∼Ub) [22]. An *in vitro* reconstitution assay for Parkin activation also indicated that Parkin E3 activity is activated by phospho-polyUb chains (S5 Figure).

**Figure 6:**
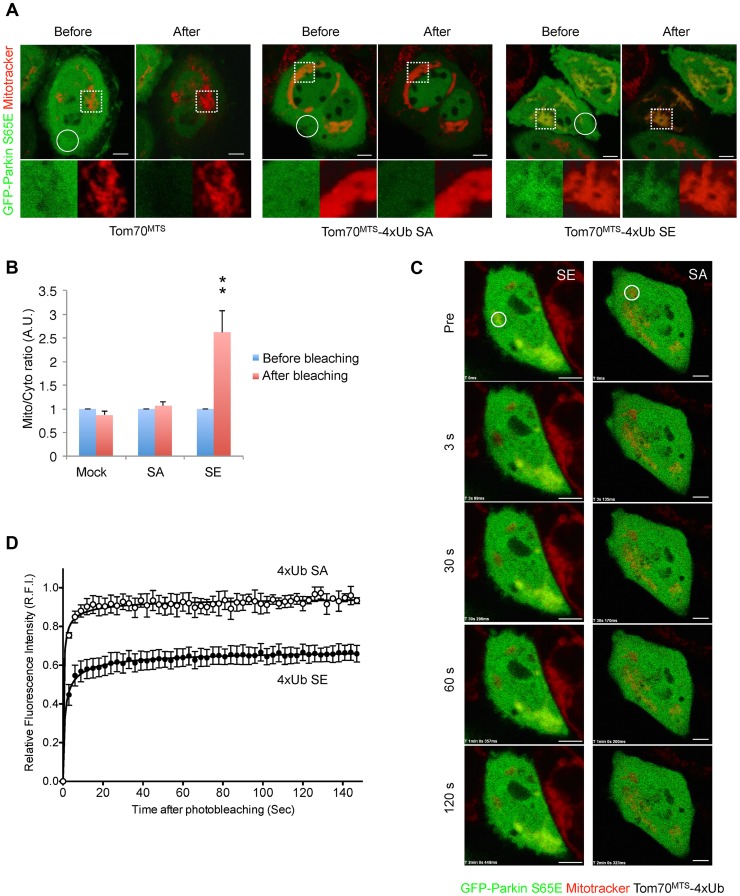
Mitochondrially targeted phospho-mimetic 4× Ub anchors Parkin. (**A**) FLIP analysis. Mitochondria of HeLa cells transfected with GFP-Parkin S65E (green) along with Tom70^MTS^, Tom70^MTS^-4Ub SA or SE were visualized with MitoTracker Red (red). Representative live cell images obtained before and after pulse photobleaching in the circled region was shown. Higher magnification images of the dashed square area were also shown in separated color channels at the bottom. (**B**) The ratios of averaged fluorescence intensity in mitochondrial and cytosolic regions (Mito/Cyto ratio, 4 µm^2^ each) were graphed (mean ±SEM, n = 4–6). ** *p*<0.01 by two-tailed unpaired Student's *t*-test. (**C**) FRAP analysis. HeLa cells transfected with GFP-Parkin WT along with Tom70^MTS^-4Ub SA or SE were treated with 2 µM a proteasome inhibitor MG132 for 3 hrs to avoid degradation of activated Parkin and mitochondria were visualized with MitoTracker Red. MG132 treatment had little effect on the molecular behavior of GFP-Parkin (see S6 Figure). Representative images of cells expressing GFP-Parkin were obtained before photobleaching (Pre) of a ROI (circle) and at the indicated times after photobleaching. (**D**) The relative fluorescence intensity (RFI) of GFP-Parkin at the mitochondria in a ROI (circle) was measured in FRAP analysis performed as in (**C**). RFI is represented as the mean ±SEM (n≥3). Scale bars  = 5 µm in (**A**, **C**).

Since Parkin was associated with and activated by phospho-polyUb chains, we determined the binding region of Parkin using Tom70^MTS^-2FLAG-4Ub SE. Parkin bound to Tom70^MTS^-2FLAG-4Ub SE through its RING1-IBR domain (Fig. 5B). Because Parkin did not show a stable binding to phospho-monoUb (Fig. 4), the interaction and activation mechanism of Parkin by the phospho-monoUb could be different from those by the phospho-polyUb chains. Given that these two factors regulate Parkin in different manners, a combination of cytosolic phospho-monoUb and mitochondrial phospho-polyUb could lead to full Parkin activation at the mitochondria. We first monitored Parkin E3 activity using Myc-Parkin C431S, with which Ub-Parkin oxyester formation can be detected after the transition to E3-activity-competent status (Fig. 5C, lane 1) [22]. SA and SE mutations were further introduced into the Ser65 position of Myc-Parkin C431S to create Myc-Parkin C431S SA and SE, respectively. Myc-Parkin C431S WT and SA do not form the Ub-Parkin oxyester bond (Parkin∼Ub) with any combination of HA-Ub SA or SE and Tom70^MTS^-2FLAG-4Ub SA or SE (Fig. 5C, lanes 2–8). In contrast, Ub oxyester formation was observed in Myc-Parkin C431S SE in the presence of HA-Ub SE, Tom70^MTS^-2FLAG-4Ub SE, or both without PINK1 accumulation, suggesting that either form of phospho-Ub is able to activate Parkin E3 (Fig. 5C, lanes 10–12 *vs.* lane 9). In this experimental setting, we did not observe obvious Mfn degradation or ubiquitination, although the Mfn1 levels tended to decrease (Fig. 5C, lanes 10–12 *vs.* lane 9). However, the application of fluorescence loss in photobleaching (FLIP) analysis in living HeLa cells expressing Tom70^MTS^-2FLAG-4Ub SE but not Tom70^MTS^-2FLAG-4Ub SA revealed that part of GFP-Parkin SE is retained on the mitochondria (Fig. 6A,B and S1-S3 Videos). A complimentary imaging analysis using fluorescence recovery after photobleaching (FRAP) technique indicated that the mobility of GFP-Parkin on the mitochondria reduced when Tom70^MTS^-2FLAG-4Ub SE but not Tom70^MTS^-2FLAG-4Ub SA was coexpressed (Fig. 6C,D and S6 Figure), suggesting that Tom70^MTS^-2FLAG-4Ub SE could stably trap GFP-Parkin at mitochondria without PINK1 activation. These imaging analyses and biochemical data supported that phosphorylated polyUb chain is required for the mitochondrial retention of Parkin.

### Mitochondrial expression of SE polyUb improves the mitochondrial degeneration in *PINK1*-deficient flies

**Figure 7:**
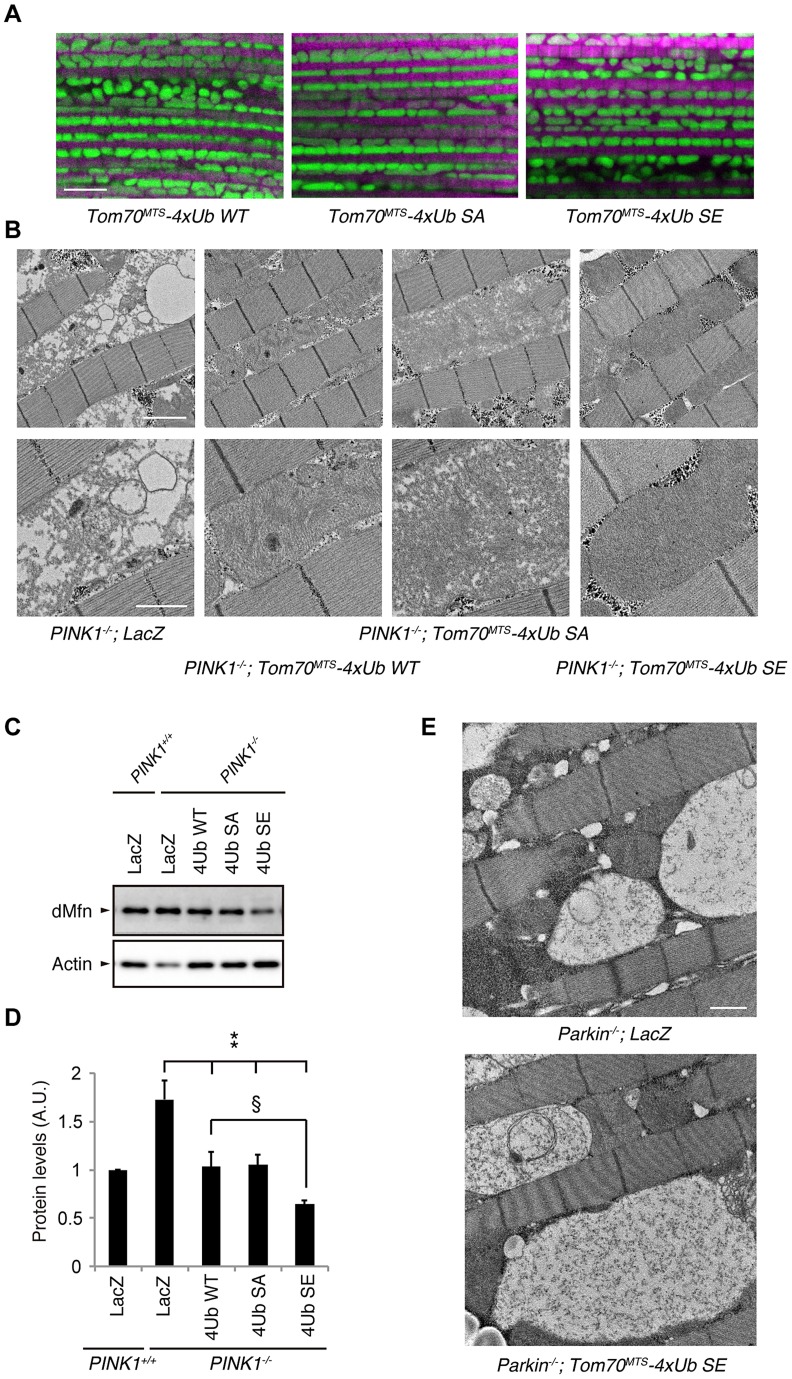
Mitochondrial expression of phospho-mimetic polyUb chain improves mitochondrial degeneration by PINK1 inactivation in *Drosophila*. (**A**) Tom70^MTS^-2FLAG-4xUb (WT, SA or SE) expression alone does not alter the mitochondrial morphology of thorax IFMs in *Drosophila*. To visualise the mitochondria, the mitoGFP (green) transgene was co-expressed, and the muscle tissue was counterstained with phalloidin (magenta). (**B**) TEM images of the IFMs in the indicated genotypes of 7-day-old adult flies are shown. (**C**) Tom70^MTS^-2FLAG-4xUb (WT, SA or SE) or LacZ was expressed in the thorax muscle of *PINK1*-deficient flies using the *MHC* driver. dMfn levels from the thoraxes of 3-day-old adult flies were analyzed by western blot. Actin served as a loading control. (**D**) The band intensities of dMfn were normalized to each actin signal shown in (**C**). The values (arbitrary units; A.U.) represent the mean ±SEM of five independent samples as in (**C**). ** *p*<0.01 *vs. PINK1^−/−^; LacZ*, § *p*<0.05. (**E**) TEM images of the IFMs in 3-day-old *Parkin*-deficient flies expressing LacZ or Tom70^MTS^-2FLAG-4xUb SE. Scale bars  = 5 µm in (**A**), 2 µm in (**B**, upper), 1 µm in (**B**, lower and **E**).

We confirmed that Tom70^MTS^-2FLAG-4Ub is also expressed in the thoracic muscle mitochondria of *Drosophila* and did not observe significant differences in the subcellular localization among Tom70^MTS^-2FLAG-4Ub WT, SA and SE (S7 Figure). The expression of Tom70^MTS^-2FLAG-4Ub showed grossly normal mitochondrial morphology in *Drosophila* thorax muscles, where mitochondrially targeted GFP (mitoGFP) was coexpressed to visualize the mitochondrial morphology and no adverse effects were detected in normal flies expressing these proteins (Fig. 7A).

We next tested whether the mitochondrial degeneration caused by PINK1 inactivation is rescued by Tom70^MTS^-2FLAG-4Ub expression, whereby endogenous *Drosophila* Parkin (dParkin) should be activated (Fig. 7B). The mitochondria of the indirect flight muscles (IFMs) are prominently affected in flies lacking PINK1 activity [4]–[6]. To examine effects of Tom70^MTS^-2FLAG-4Ub expression on the mitochondrial morphology of IFMs in detail, transmission electron microscopy (TEM) analysis was performed in *PINK1^−/−^* flies expressing a control LacZ and Tom70^MTS^-2FLAG-4Ub WT, SA or SE (Fig. 7B). Mitochondrial swelling and matrix disorganization were observed in the *PINK1^−/^* ^−^ *; LacZ* flies, which was partly rescued by Tom70^MTS^-2FLAG-4Ub WT or SA expression (Fig. 7B). The electron density of mitochondrial matrices was recovered to normal levels by the introduction of Tom70^MTS^-2FLAG-4Ub SE (Fig. 7B).

dParkin is thought to be inactive in the absence of dPINK1 activity. Thus, the accumulation of dMitofusin (dMfn), which is one of the substrates of dParkin, is observed in *PINK1*-deficient flies [32], [33]. We examined the dMfn levels in the thorax muscles to estimate endogenous dParkin activity in *PINK1* knockout flies expressing LacZ, Tom70^MTS^-2FLAG-4Ub WT, SA or SE (Fig. 7C,D and S8 Figure). There was a tendency toward decreased dMfn levels with Tom70^MTS^-2FLAG-4Ub WT or SA, and Tom70^MTS^-2FLAG-4Ub SE significantly decreased dMfn, suggesting that endogenous dParkin is efficiently activated by phosphorylated Ub chains in *Drosophila* (Fig. 7C, D). In a similar setting, the expression of Tom70^MTS^-2FLAG-4Ub SE failed to rescue the mitochondrial degeneration in *Parkin* knockout flies, implying that the mitochondrial expression of phospho-polyUb does not activate other selective autophagy pathways to compensate the PINK1-Parkin pathway (Fig. 7E).

**Figure 8:**
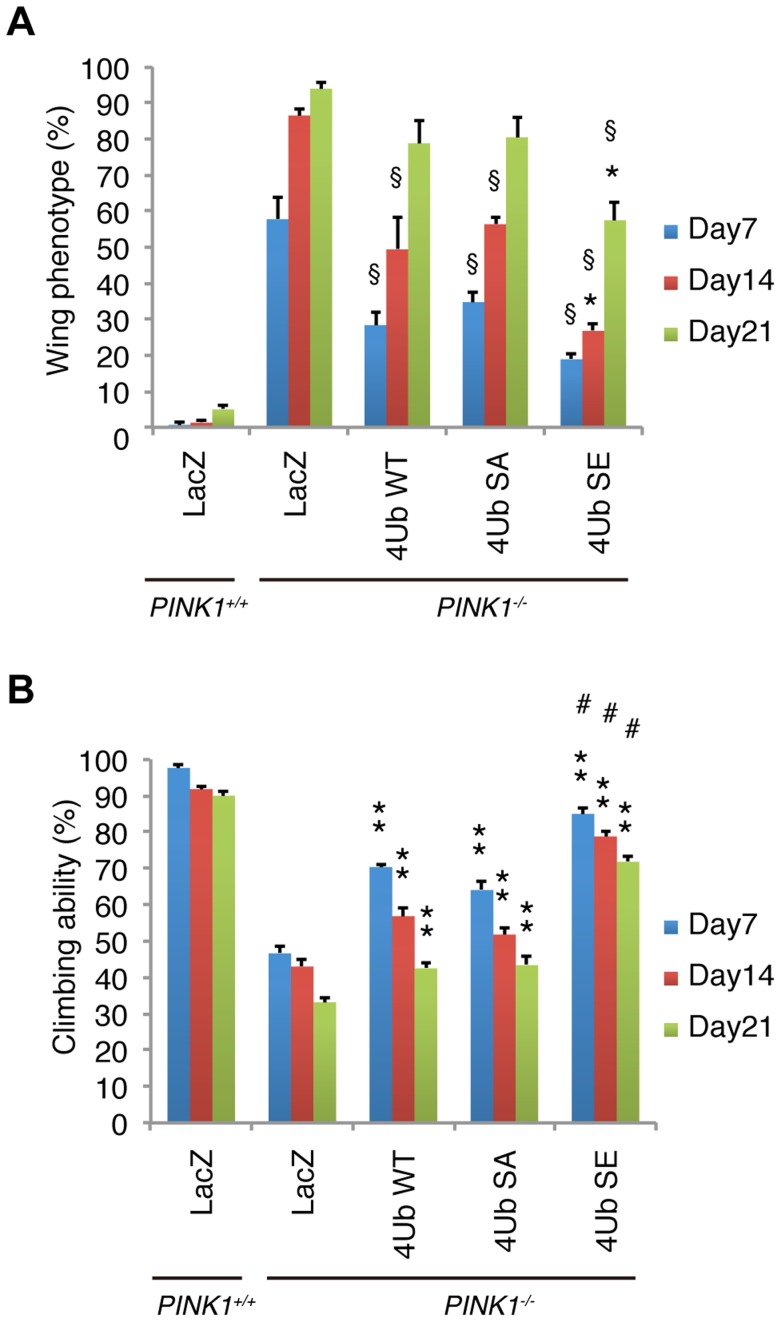
Mitochondrial phospho-mimetic polyUb chain improves the PINK1 phenotypes in *Drosophila*. (**A**) The mitochondrial expression of 4xUb (WT, SA and SE) rescues the abnormal wing posture caused by the loss of *PINK1* activity (§ *p*<0.01, *LacZ vs. WT, SA* or *SE in PINK1^−/−^* at the same age), and 4xUb SE exerted a more profound effect (* *p*<0.05, *SE vs. WT* or *SA in PINK1^−/−^* at the same age). LacZ or Tom70^MTS^-2FLAG-4xUb (WT, SA or SE) was expressed under the control of *MHC-GAL4* in the *w-* or the *PINK1^−/−^* backgrounds. LacZ served as a control. Graph showing the percentage of flies with abnormal wing posture. Adult male flies aged 7, 14 and 21 days were analyzed. *n* = 159–168. (**B**) The mitochondrial expression of 4xUb (WT, SA and SE) improved the motor activity throughout the trials (** *p*<0.01, *LacZ vs. WT, SA* or *SE in PINK1^−/−^* at the same age), whereas 4xUb SE showed the best motor activity in every trial (# *p*<0.01, *SE vs. WT* or *SA in PINK1^−/−^* at the same age). The values are presented as the mean ±SEM of 20 trials. Male flies were used for the assay.

The expression of any form of Tom70^MTS^-2FLAG-4Ub significantly suppressed the abnormal wing posture caused by the mitochondrial degeneration in the IFMs of 7-day-old adult flies (Fig. 8A). However, the suppression effects of Tom70^MTS^-2FLAG-4Ub WT and SA gradually reduced and showed little difference from the control LacZ in 21-day-old flies (Fig. 8A). In contrast, Tom70^MTS^-2FLAG-4Ub SE suppressed the wing abnormalities more efficiently throughout the analysis, although ageing weakened the suppression effects (Fig. 8A). Similar to the results obtained in the wing phenotype, the climbing defects caused by PINK1 inactivation were improved by the expression of all three types of Tom70^MTS^-2FLAG-4Ub, with Tom70^MTS^-2FLAG-4Ub SE having the strongest effect (Fig. 8B). The phenotypic and behavioural analyses suggested that endogenous Parkin expression could be activated by the mitochondrial expression of phospho-polyUb chains.

## Discussion

A series of *Drosophila* genetic and cell biological studies have revealed that PINK1 is required for Parkin-mediated mitochondrial maintenance. The mitophagy of damaged mitochondria is thought to involve PINK1 and Parkin. Most of the mutations in PINK1 and Parkin that are found in PD affect the process of mitophagy, which suggests that dysfunction in the mechanism for eliminating damaged mitochondria is part of the etiology of PD, and this understanding of PINK1/Parkin-mediated mitochondrial maintenance will be helpful in the prevention of neurodegeneration in PD. However, how PINK1 recruits Parkin to the damaged mitochondria in the mitochondrial quality control remains unclear.

To identify the mitochondrial translocation factor(s) of Parkin, we searched for PINK1 substrate(s) using whole cells treated with or without CCCP and found that Ub at Ser65 was specifically phosphorylated by PINK1 activation. During preparation of this manuscript, three groups independently reported that PINK1 phosphorylates Ub to activate Parkin E3 activity [25]–[27]. Our study further reveals that the phospho-polyUb chain is implicated in Parkin translocation, predicting a chain reaction step of Parkin activation and translocation.

**Figure 9:**
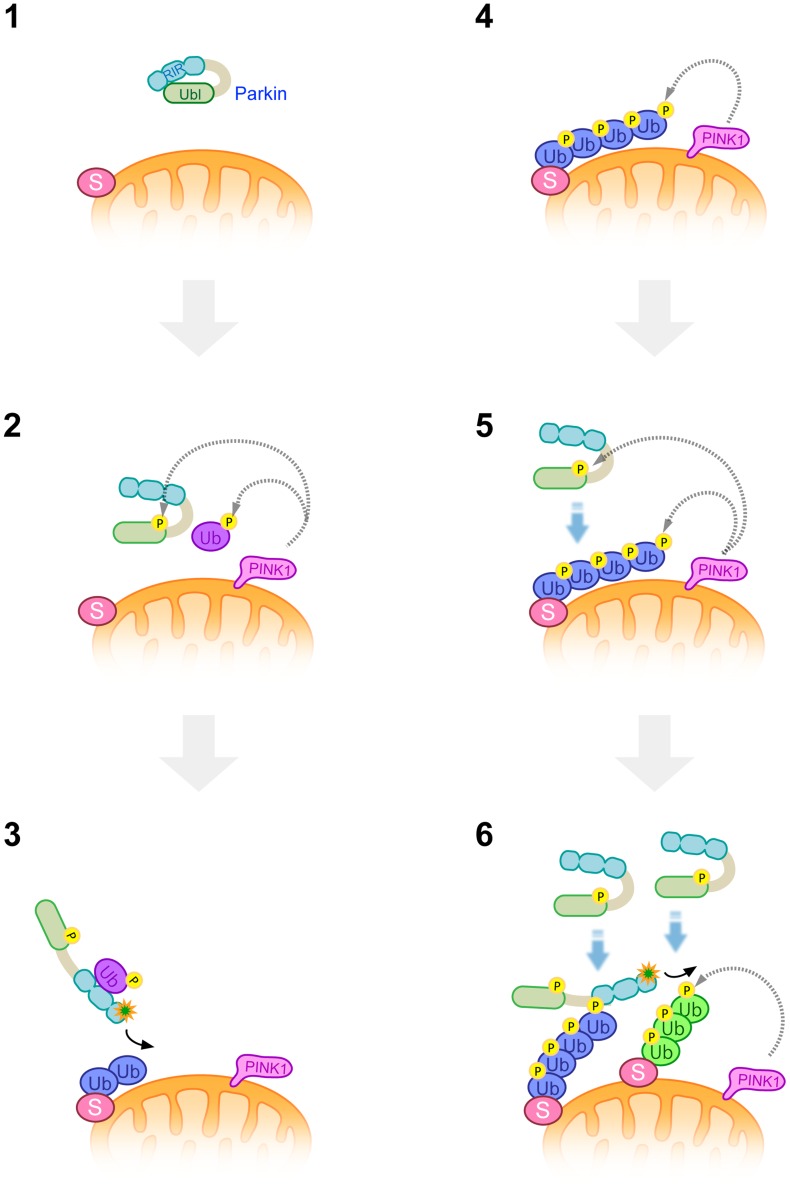
A model of Parkin E3 activation and mitochondrial translocation. A latent form of cytosolic Parkin (**1**) and Ub adjacent to the mitochondrial membrane is phosphorylated by PINK1 upon depolarization in ΔΨm (**2**). Phosphorylation of the Parkin Ubl domain weakens the masking effect on its catalytic domain (**2**). Intramolecular association of the Ubl domain with the catalytic domain is replaced by that of phosphorylated Ub, or phosphorylated Ub induces a conformational change in Parkin, whereby another translocation promoting factor might bind to the Parkin linker region, leading to the mitochondrial translocation of Parkin and substrate (S) ubiquitination (**3**). K48-linked and K63-linked polyubiquitinated chains attached to the substrate are also phosphorylated by PINK1 (**4**). The remaining Parkin in the cytosol preferentially associates with K63-linked phosphorylated polyUb chains (**5**) and amplifies the Parkin activation cascade, generating additional phosphorylated polyUb chains in cooperation with PINK1 (**6**).

A previous study suggested that Parkin binds to K63-linked polyUb *in vitro* and that their binding is potentiated in the presence of PINK1 [29]. Supporting this finding, our study demonstrated that Parkin preferentially binds to K63-linked polyUb chains phosphorylated at Ser65 by PINK1, which is involved in Parkin activation and stable mitochondrial localization. From our results, the existence of an amplification reaction of Parkin mitochondrial tethering is conceivable (Fig. 9 and S9 Figure). Upon reduction of ΔΨm, PINK1 is activated and phosphorylate Ub adjacent to the mitochondrial membrane. A small part of cytosolic Parkin activated by phospho-monoUb initially generates polyUb chains on the mitochondria probably through its substrates [22]. PINK1 phosphorylates Ub chains generated by Parkin, whereby the remaining cytosolic Parkin binds to and is activated by phosphorylated Ub chains, generating newly phosphorylated Ub chains in cooperation with PINK1 (Fig. 9 and S9 Figure). Although both K63-linked and K48-linked phospho-polyUb chains are generated in this context, Parkin pull-down assay using two kinds of polyUb chains revealed that Parkin isolated form cultured cells exhibited a more affinity to longer K63-linked phospho-polyUb chains, suggesting that K63-linked phospho-Ub chain mainly contributes to both Parkin activation and translocation. K48-linked phospho-Ub chain might be used for the proteasomal recognition and degradation of Parkin substrates, activating Parkin E3. Currently, we cannot completely exclude the possibility that long forms of K48-linked phospho-polyUb also contribute to mitochondrial recruitment of Parkin since a weaker association of Parkin with K48-linked phospho-polyUb_3-7_ was also detected.

Parkin purified from bacteria bound to both phospho-polyUb chains. The result might suggest the existence of a factor to determine the preferential binding of Parkin to K63-linkage specific polyUb chain, which could be a protein(s) co-purified from cultured cells or could be an unknown modification of Parkin itself.

The involvement of K27-linked polyUb formation has also been suggested during Parkin-mediated mitophagy. Thus, the possible contribution of K27-linked phospho-Ub remains to be solved [8]. A recent study has reported that Parkin is involved in the formation of linear Ub chain, activating NF-κB signaling [34]. HOIL-1 is an accessory protein of the linear Ub chain assembly complex, which is a key factor for efficient formation of the linear Ub chains [35]. Efficiency of Parkin mitochondrial translocation was similar in the presence or absent of HOIL-1 (S10 Figure), suggesting that the linear Ub chain is not involved in Parkin recruitment.

However, the combination of Parkin SE and Tom70^MTS^-4Ub SE did not fully recapitulate rapid Parkin translocation as observed in CCCP- or valinomycin-treated cells and PINK1 activity was still required for effective translocation of Parkin, implying that an unknown factor(s) regulated by PINK1 might be missing. Alternatively, Tom70^MTS^-4Ub SE might not fully mimic phospho-K63-linked polyUb chain on the mitochondria and the mechanism for exponential K63-linked phospho-Ub production in cooperation with activated PINK1 might be required.

Our study also indicates that the phospho-polyUb chain binds to the RING1-IBR region and predicted that another translocation factor(s) is involved through an association with the Parkin linker region containing RING0 because the RING-IBR-RING mutant does not translocate to mitochondria accumulating phospho-polyUb and because ΔUbl Parkin itself has mitochondrial translocation activity [24]. The predicted protein that interacts with the Parkin linker region could be the above-mentioned missing factor(s). Our results explain why mutations in multiple domains of Parkin affect its mitochondrial translocation. A recent study suggested that Parkin self-associates through the RING-IBR-RING domain upon CCCP treatment. In this experimental setting, E3-dead Parkin was recruited to the RING-IBR-RING mutant tethered to mitochondria with CCCP treatment, suggesting that cytosolic Parkin binds to mitochondrial Parkin without E3 activation once Parkin is recruited to the mitochondria [22]. Another explanation of this result from our findings (Fig. 3) is that PINK1-dependent phospho-polyUb could be generated on the mitochondria upon CCCP treatment because RING-IBR-RING has potent E3 activity, thus recruiting E3-dead Parkin to phospho-polyUb [30]. Further studies will clarify which molecular mechanisms are involved in the amplification step of Parkin translocation.

Our *Drosophila* study revealed that the mitochondrial expression of a phospho-mimetic polyUb chain (Tom70^MTS^-2FLAG-4Ub SE) stimulates endogenous Parkin activation in the absence of PINK1, as shown by the reduced levels of the Parkin substrate dMfn and the improvement of mitochondrial morphology and related phenotypes. In contrast to the rescue effects of ectopic Parkin expression on *PINK1*-mutant flies, the effects of Tom70^MTS^-2FLAG-4Ub SE were influenced by ageing. This observation suggests that endogenous Parkin expression decreases or that Parkin inhibition factors accumulate with age. Supporting this idea, the age-dependent accumulation of dMfn has been reported [36].

Although we could not show whether endogenous dParkin is also relocated to mitochondria by Tom70^MTS^-2FLAG-4Ub SE in *Drosophila* due to the absence of appropriate antibodies, a previous study reported that overexpressed Parkin is localized at mitochondria [5]. Thus the mitochondrial expression of 4xUb might contribute to Parkin E3 activation as shown in S5 Figure rather than the mitochondrial localization of Parkin in *Drosophila*.

The expression of Tom70^MTS^-2FLAG-4Ub WT and SA, both of which should be non-phosphorylated in the *PINK1*-mutant flies, also alleviated the *PINK1*-mutant phenotypes albeit less than that of Tom70^MTS^-2FLAG-4Ub SE. The results appear to reflect the observation that Tom70^MTS^-2FLAG-4Ub SA partially recruited E3-dead form of Parkin (C431S) to the mitochondria in cultured cells (Fig. 2B), which might be attributed to an unknown Parkin regulator(s) recognizing the mitochondrial polyUb independently of the phosphorylation status. Alternatively, the expression of mitochondrial polyUb might activate another pathway for mitochondrial maintenance, partially compensating for the mitochondrial quality control by the PINK1-Parkin signalling although the rescue effect disappeared in the *Parkin*-deficient flies.

In conclusion, our data support the possibility that there are elaborate multi-amplification steps for the mitochondrial translocation of Parkin by PINK1 activation (Fig. 9 and S9 Figure) involving the PINK1-mediated phosphorylation of Ub and Parkin. This study also characterizes Parkin as an E3 ligase that recognizes the K63-linked phospho-polyUb chain. Although PINK1 is the first reported kinase for Ub, the existence of another Ub kinase(s) regulated by a variety of stressors would explain why Parkin protects cells from various stress conditions, regulating many different substrates.

### Note

During revision of this manuscript, a study from the Harper lab [37] proposed a similar amplification model for Parkin mitochondrial translocation and activation, in which they reported that phospho-Parkin specifically binds to both phospho-K63 and phospho-K48-linked polyUb chains *in vitro*, a consistent result with ours (Fig. S4). Our recent study suggested that the reduction of K63-linked polyUb formation does not affect Parkin-mediated mitophagy [38]. Considering these data, other phospho-polyUb including K48-linkage might contribute to Parkin activation sufficiently.

## Materials and Methods

### Antibodies and plasmids

The rabbit anti-dMfn polyclonal antibody was raised against a mixture of synthetic peptides (C-DTVDKSGPGSPLSRF and C-IQNELDIFEHNYISPQ) and affinity-purified (Japan Bio Services). The following antibodies were used in the western blot analysis: anti-PINK1 (1∶1,000 dilution, Novus, BC100–494; or 1∶1,000 dilution, Cell Signaling Technology, clone D8G3), anti-dMfn (1∶2,000 dilution; made in-house), anti-Mfn1 (1∶1,000 dilution; Abnova, clone 3C9), anti-VDAC1 (1∶1,000 dilution; Abcam, Ab15895), anti-Tom20 (1∶500 dilution; Santa Cruz Biotechnology, FL-145), anti-FLAG-HRP (1∶2,000 dilution; Sigma-Aldrich, clone M2), anti-GFP (1∶1,000 dilution; Wako, clone mFX75), anti-Ub (1∶5,000 dilution; made in-house, Ub2), anti-Actin (1∶10,000 dilution; Millipore, MAb1501) and anti-Hsp60 (1∶10,000 dilution, BD Biosciences, clone 24/Hsp60). The following antibodies were used for immunocytochemistry: anti-FLAG (1∶1,000 dilution; Sigma-Aldrich, clone M2), anti-Parkin (1∶1,000 dilution; Cell Signaling Technology, clone PRK8), anti-polyUb (1∶250 dilution; MBL, clone FK2), anti-Tom20 (1∶1,000 dilution; Santa Cruz Biotechnology, FL-145) and anti-Myc (1∶500 dilution; Millipore, clone 4A6). The cDNAs encoding Ub, human Parkin, PINK1 and the pathogenic and engineered mutants are as described in previous studies [9], [39], [40]. Tom70^MTS^-2FLAG-4Ub was a kind gift of Drs X. Zheng and T. Hunter [29]. Ub phospho-mutants were newly generated by PCR-based mutagenesis followed by sequencing confirmation of the entire gene. For Tom70^MTS^-RBR, the 2FLAG-4Ub region of Tom70^MTS^-2FLAG-4Ub was replaced with the Parkin RBR. Complementary DNA encoding dParkin SE was described in a previous study [41].

### Cell cultures

*PINK1^−/−^* MEFs or *Ubc^−/−^* MEFs, cultured as previously described [9], were retrovirally transfected with pMXs-puro harbouring PINK1-FLAG or GFP-Parkin, and the transfected cells were then selected with 1 µg/ml puromycin. HeLa and HEK293T cells were maintained at 37°C with a 5% CO_2_ atmosphere in DMEM (Sigma-Aldrich) supplemented with 10% FCS (Gibco), GlutaMax (Gibco), non-essential amino acids (Gibco) and 1% penicillin-streptomycin. HeLa cells stably expressing non-tagged Parkin were described previously [24]. *PINK1^−/−^* HeLa cells and *HOIL-1^−/−^* MEFs were kind gifts from Drs R. Youle [42] and K. Iwai [35], respectively. The plasmids were transfected using Lipofectamine 2000 (Life Technologies) or Lipofectamine Plus (Life Technologies), and the siRNA duplexes (Life Technologies) were transfected using Lipofectamine RNAiMAX (Life Technologies), according to the manufacturer's instructions. The total amount of transfected cDNA was adjusted with vector DNA in every transfection experiment.

### Analysis of protein phosphorylation by mass spectrometry

We prepared two sets of samples for screening the PINK1 substrates: (1) CCCP-treated PINK1-FLAG WT/*PINK1^−/−^* MEFs treated with 30 µM CCCP for 30 min *vs.* CCCP-treated PINK1-FLAG KD/*PINK1^−/−^* MEFs treated with 30 µM CCCP for 30 min and (2) CCCP-treated PINK1-FLAG WT/*PINK1^−/−^* MEFs treated with 30 µM CCCP for 30 min *vs.* CCCP-treated PINK1-FLAG WT/*PINK1^−/−^* MEFs with DMSO treatment. The cell pellets (1.0×10^7^) were lysed with 8 M urea buffered with 50 mM Tris-HCl at pH 9.0 and digested with trypsin. Stable isotope dimethyl labelling with ^13^CD_2_O or ^12^CH_2_O [43] was performed for each set of samples. After mixing the differentially labelled samples, relative quantitation was conducted by nano-scale liquid chromatography-tandem mass spectrometry (Thermo Ultimate3000 RSLCnano and ABSciex TripleTOF 5600) followed by MASCOT searching and Mass Navigator/PhosPepAnalyzer processing [44]. The determination of phosphosite localization was performed based on the presence of site-determining ions [45].

### Ub phosphorylation and Parkin activation assays

Recombinant glutathione S-transferase (GST), GST-fusion Ub, GST-fusion Parkin Ubl domains, maltose-binding protein (MBP) fusion-human Parkin and His_6_-SUMO-Parkin were bacterially produced and then purified by affinity chromatography with GSH beads (GE healthcare), amylose beads (New England Biolabs) or Ni-NTA beads (Qiagen). His_6_-SUMO-Parkin was cleaved using His_6_-SENP1 Catalytic Domain (Boston Biochem) to obtain non-tagged Parkin as described [15]. Recombinant His-Ub and K48- and K63-linked polyUb were purchased from Boston Biochem. Recombinant MBP-*T. castaneum* PINK1 (TcPINK1) purified from bacteria was used as a kinase source [46], and *in vitro* kinase assays using autoradiography and phos-tag were performed as described [24]. For Parkin activation assay, 6.6 µM human Ube1, 16.7 µM human UbcH7, and 16.7 µM FLAG-Ub (Sigma-Aldrich) in Ub buffer (20 mM HEPES, pH 7.5, and 50 mM NaCl) in the presence of 2 mM MgCl_2_ and 2 mM ATP were incubated for 60 min at 30°C, which were divided into 5 µl aliquots. Linkage specific recombinant polyUb_3–7_ chains (Boston Biochem) were phosphorylated by either MBP-TcPINK1 WT or KD in parallel. Phosphorylation efficiency was confirmed by Phos-tag western blot using anti-Ub. After removal of MBP-TcPINK1 with amylose resin, 1 µg of polyUb and 1 µg of MBP-human Parkin in 15 µl of Ub buffer were combined with the above mixture and further incubated for 15 min at 30°C. Reactions were terminated by the addition of 10 µl of 3×SDS loading buffer without reducing reagents.

### Parkin pull-down assay

FLAG-Parkin was immunopurified from HEK293T cells expressing FLAG-Parkin using anti-FLAG M2 beads (Sigma-Aldrich). Bovine Ub (2 µg, Sigma-Aldrich) and recombinant K48- and K63-linked polyUb (10 µg each) were phosphorylated by MBP-TcPINK1 (WT and KD, 16 µg each) at 30°C for 120 min in *in vitro* kinase buffer as described [24]. After removal of MBP-TcPINK1 with amylose beads, polyUb was further incubated with FLAG-Parkin (∼200 ng, estimated by bands stained with CBB) conjugated anti-FLAG beads or anti-FLAG beads alone as a mock control in a pull-down buffer (20 mM Tris-HCl, pH7.5, 200 mM NaCl, 1 mM EDTA, 1 mM DTT) for 60 min at 4°C. FLAG-Parkin was then eluted with the pull-down buffer containing 100 µg/ml of FLAG peptide.

For the assay using recombinant Parkin from bacteria, 600 ng of non-tagged Parkin was incubated with the polyUb treated as above. Parkin was immunoprecipitated with anti-Parkin (PRK8) conjugated Protein G beads (GE healthcare) at RT for 30 min. After washing three times, Parkin was eluted with 0.2 M glycine-HCl (pH 2.5) at 4°C for 60 min with gentle shaking.

### Immunocytochemical and western blot analyses

Cells plated on 3.5-mm glass-bottom dishes (MatTek) were treated with or without 10 µM CCCP or valinomycin and were stained with the indicated antibodies as described [24]. The cells were imaged using laser-scanning microscope systems (TCS-SP5, Leica or LSM510 META, Carl Zeiss). Western blot analysis using cultured cells and *Drosophila* tissue samples was performed as described, using ECL prime solution (GE Healthcare) [24], [41]. The blot images were obtained with X-ray film or Image Quant LAS 4000 mini (GE Healthcare).

### FLIP and FRAP analyses

Live cell imaging was performed using an Olympus FV-1000 inverted confocal microscope equipped with a 60× oil lens (NA = 1.42). For FLIP and FRAP analyses, cells were placed on a stage top incubator (Tokai Hit) maintained at 37 °C supplied with 5% CO_2_. For FLIP analysis, a cytoplasmic region was photobleached using a 404-nm wavelength laser with 100% laser power for 2 sec with 8 sec intervals in a ROI (3 µm diameter). Dual fluorescence (GFP and MitoTracker Red) images were collected sequentially at 4× zoom power every 10 sec after pulse-photobleaching for a total of 100 sec. For FRAP analysis, each clustered mitochondrial region was photobleached using a 404-nm wavelength laser with 100% laser power for 100 msec in a ROI (2 µm diameter). Single-scanned images were collected at 4× zoom power every 3 sec for a total of 150 sec. Relative fluorescence intensities (RFI) were calculated based on measurement of the fluorescence intensity in the photobleached area normalizing with that in an adjacent cytoplasmic area. GraphPad Prism software was used to fit all curves. Curve fit analysis was performed using the following equation (eq1), which is commonly used for FRAP data fitting and the determination of diffusion constants [47].

I(t)  = YMAX(1-w^2^(w^2^+4πDt)^−1^)^1/2^ (1)

Parameters are defined as follows; I(t) represents a fluorescence intensity as a function of time, YMAX is a final intensity reached after complete recovery, w^2^ is an area of the photobleaching i.e. 3.14 µm^2^, and D is an effective one-dimensional diffusion constant. The values of YMAX and D were calculated by fitting this function to the experimental data. YMAX and D represent the ratio of immobile and mobile fractions in a photobleaching area and the dynamics of fluorescent molecules, respectively.

### *Drosophila* genetics

Fly culture and crosses were performed on standard fly food containing yeast, cornmeal and molasses, and the flies were raised at 25°C. The *w^1118^* (*w^–^*) line was used as the WT genetic background. Complementary DNAs encoding Tom70^MTS^-2FLAG-4Ub WT, SA and SE were subcloned into the *pUAST* vector, and *UAS- Tom70^MTS^-2FLAG-4Ub WT, SA* and *SE* transgenic lines were generated in the *w^–^* background (BestGene). All other fly stocks and *GAL4* lines used in this study were obtained from the Bloomington *Drosophila* Stock Center and have been previously described: *PINK1^B9^* [5], *Parkin* ^Δ*21*^ [48] and *Parkin^1^* [49]. *PINK1^B9^* and *Parkin* ^Δ*21*^/Parkin^1^ flies were used as *PINK1-*deficient and *Parkin*-deficient alleles, respectively.

The genotypes used in this study were as follows: (Fig. 7A) *UAS-mitoGFP/UAS-Tom70^MTS^-2FLAG-4xUb WT; MHC-GAL4 (4xUb WT)*, *UAS-mitoGFP; MHC-GAL4/UAS-Tom70^MTS^-2FLAG-4xUb SA (4xUb SA)*,* UAS-mitoGFP; MHC-GAL4/UAS-Tom70^MTS^-2FLAG-4xUb SE (4xUb SE)*, (Fig. 7B and Fig. 8A,B) *PINK1^B9^/Y; UAS-LacZ; MHC-GAL4 *(*PINK1^−/−^; LacZ*),* PINK1^B9^/Y; UAS- UAS-Tom70^MTS^-2FLAG-4xUb WT*;* MHC-GAL4 *(*PINK1^−/−^; Tom70^MTS^-4xUb WT*);* PINK1^B9^/Y; +/+*;* MHC-GAL4/UAS-Tom70^MTS^-2FLAG-4xUb SA *or *SE *(*PINK1^−/−^; Tom70^MTS^-4xUb SA *or *PINK1^−/−^; Tom70^MTS^-4xUb SE*),* UAS-LacZ*;**and *MHC-GAL4 *(*PINK1^+/+^; LacZ*), (Fig. 7E) *UAS-LacZ*;* Da-GAL4, Parkin* ^Δ*21*^ */Parkin^1^*(*Parkin^−/−^), UAS-Tom70^MTS^-2FLAG-4xUb SE*;* Da-GAL4, Parkin* ^Δ*21*^ */Parkin^1^*(*Parkin^−/−^; Tom70^MTS^-4xUb SE*).

### Whole-mount immunostaining and TEM analysis

The mitochondrial morphology of the IFMs was analyzed by whole-mount immunostaining as described previously [50]. For the mitochondrial imaging in Fig S7C, dissected thorax muscles were cultured in Schneider's medium (Gibco) containing 200 nM MitoTracker Red CMXRos (Molecular Probes) for 30 min at RT and were washed with Schneider's medium three times before fixation. TEM images were obtained at the Laboratory of Ultrastructural Research of Juntendo University.

### Quantification of wing phenotypes and climbing assay

To control for isogeny, the driver and *PINK1^B9^* lines were backcrossed to the *w^−^* WT background for six generations. All transgenic flies were generated in the *w^−^* genetic background and thus have matched genetic backgrounds. The number of flies exhibiting defective, abnormal wing posture (held up or drooped) was determined for each genotype [6]. A climbing assay was performed as described previously [51].

### Statistical analysis

A one-way repeated measures ANOVA was used to determine significant differences between multiple groups unless otherwise indicated. If a significant result was achieved (*p* <0.05), the means of the control and the specific test group were analyzed using the Tukey-Kramer test.

## Supporting Information

Figure S1 Phosphorylation of Ser65 in Ub and Parkin activate Parkin's E3 activity in an additive manner. (**A**) The autoubiquitination of GFP-Parkin is stimulated by Ub. Lysates from HEK293T cells transfected with the indicated combinations of cDNA were analysed by western blotting. The mitochondrial matrix protein Hsp60 served as a loading control. The expression of HA-Ub WT tended to reduce Parkin levels, suggesting that WT Ub is used for Parkin degradation rather than activation. (**B**) The coexpression of GFP-Parkin SE with HA-Ub SE does not stimulate the mitochondrial translocation of Parkin in HeLa cells. GFP-Parkin and mitochondria were visualized with GFP signal (green) and anti-Tom20 (red), respectively. Scale bar  = 10 µm.
(TIF)

Figure S2 Reduction of Ub expression attenuates Parkin mitochondrial translocation. (**A**) Ub is required for Parkin translocation. HeLa cells stably expressing non-tagged Parkin were transfected with mock siRNA or an siRNA mixture for RPS27A, UBA52, UBB and UBC (2.5 nM each, Silencer, Life Technologies). At 48 hr post-transfection, the cells were treated with 10 µM CCCP for 30 min. Parkin and the mitochondria were visualized with anti-Parkin (green) and anti-Tom20 (red), respectively. The Parkin and Tom20 signals are also shown as monochrome images. (**B, C**) The reduced expression of Ub delays Parkin translocation to the depolarized mitochondria. (**B**) *Ubc^+/+^* or *Ubc^−/−^* MEFs retrovirally introduced with GFP-Parkin were treated with 30 µM CCCP for 4.5 hr. GFP-Parkin and the mitochondria were visualized with the GFP signal (green) and anti-Tom20 (red), respectively. The Parkin and Tom20 signals are also shown as monochrome images. (**C**) The GFP signals colocalized with anti-Tom20 as in (**B**) were extracted using ImageJ. The mitochondrial translocation efficiency is presented by the percentage of cells with the mitochondrial GFP signals over 2-fold median fluorescence intensity in each image. The graph shows the means ±SEM in three independent experiments, with ∼90 cells counted per sample. *** *p*<0.001 (two-tailed unpaired Student's *t*-test). Scale bars  = 10 µm in (**A**), 30 µm in (**B**).
(TIF)

Figure S3 Neither mitochondrial monoUb nor cytosolic polyUb promotes Parkin recruitment. (**A**) Mitochondrial phospho-monoUb does not recruit Parkin C431S efficiently. The graph indicates means ±SEM of the percentages of cells exhibiting mitochondrial recruitment in three independent experiments. 1xUb WT, SA or SE *vs.* 4Ub WT, **p<0.01; N.S., not significant by the Tukey-Kramer test. (**B**) PINK1 is required for Parkin C431S recruitment by Tom70^MTS^-4xUb. A similar experiment as in Fig. 2A was performed using normal HeLa cells (PINK1^+/+^) and *PINK1*-deficient HeLa cells (PINK1^−/−^). Parkin WT was also included as controls. Scale bar  = 10 µm. (**C, D**) Cytosolic 4xUb SE does not recruit Parkin C431S efficiently and Tom70^MTS^-4xUb SE fails to recruit Parkin WT to mitochondria in the absence of PINK1. HeLa cells (PINK1^+/+^) expressing GFP-Parkin C431S along with Tom70^MTS^-4xUb SE or 4xUb SE without Tom70^MTS^ (Cyto-4xUb SE) and *PINK1*-deficient HeLa cells (PINK1^−/−^) expressing GFP-Parkin WT along with Tom70^MTS^-4xUb SE were treated as shown in Fig. 2. (**C**) The graph indicates means ±SEM of the percentages of cells exhibiting mitochondrial recruitment in three independent experiments, with ∼100 GFP-positive cells counted per sample. Cyto-4xUb SE *vs.* Tom70^MTS^-4xUb SE, ***p<0.001 (two-tailed unpaired Student's *t*-test). (**D**) Representative images as in (**C**). Scale bar  = 10 µm.
(TIF)

Figure S4 Parkin produced in bacteria binds to both K48-linked and K63-linked phospho-Ub chains. (**A**) Non-tag Parkin prepared from bacteria was visualized by CBB staining (left). K48-linked and K63-linked polyUb_3–7_ used in this study (right). (**B**) *In vitro* Parkin pull-down assay for linkage-specific phospho-Ub chains was performed as in Fig. 4.
(TIF)

Figure S5 Parkin is activated by both K48-linked and K63-linked phospho-Ub chains. MBP-human Parkin WT and Ser65A (SA) were incubated for 15 min at 30°C with K48-polyUb_3–7_ or K63-polyUb_3–7_ pretreated with TcPINK1 WT or KD. Control reactions without MBP-Parkin, polyUb or incubation at 30°C were also performed through the same procedure. Dots indicate putative Ube1∼Ub bands. Phosphorylation of Ub chains (asterisks) was confirmed by Phos-tag western blot (Left). Note that Phos-tag polyacrylamide gel electrophoresis is sensitive to the conformation and charge of proteins, and does not always reflect their actual molecular mass.
(TIF)

Figure S6 Mitochondrial phosphomimetic 4× Ub reduces the mobility of GFP-Parkin localized at the mitochondria. Quantitative FRAP analysis was performed as in Fig. 6 in the presence or absence of 2 µM MG132. RFI is represented as the mean ±SEM (n≥3). The values of D and YMAX indicate that Tom70^MTS^-4xUb SA does not recruit GFP-Parkin at the mitochondria (Mito) and MG132 treatment does not affect the diffusion of GFP-Parkin in the cytoplasm (Cyto).
(TIF)

Figure S7 Expression of Tom70^MTS^-2FLAG-4Ub in the *Drosophila* thorax. (**A**) The levels of *Tom70^MTS^-2FLAG-4Ub* transcripts were measured using quantitative RT-PCR and were normalized by housekeeping *rp49* levels. Total RNA was extracted from the thoraxes of 5-day-old adult male flies (n = 10). All measurements were performed in triplicate, and values represent the means ±SEM. Expression of 4Ub SE was reduced compared with 4Ub SA (*p*<0.01 by the Tukey-Kramer test) and there were no significant differences between other combinations. (**B**) The protein levels of Tom70^MTS^-2FLAG-4Ub in the thoraxes of 5-day-old adult male flies were examined using Western blot. Actin signal served as a loading control. Red dots and asterisks indicate putative endogenous Ub modifications and non-specific bands detected by the antibody. (**C**) Mitochondrial polyUb signals in Tom70^MTS^-2FLAG-4Ub-expressing flies. Fluorescent images of the indirect flight muscle in the indicated genotypes of 5- to 7-day-old adult flies are shown. LacZ expression was used as a control. PolyUb signals (green) were visualized with anti-polyUb antibody (FK2, 1∶100) and the muscle tissues were counterstained with MitoTracker (red) and phalloidin (blue). Scale bar  = 5 µm. Expression of the transgenes was induced by the *MHC*-*GAL4* driver in (**A–C**).
(TIF)

Figure S8 Specificity of anti-dMfn antibody. *UAS-dMfn* (a kind gift from Dr M. Feany) and *UAS-dMfn RNAi* (VDRC stock) were driven by *MHC-GAL4*. *MHC-GAL4* crossed with *w-* was used as a normal control (Normal). Muscle tissues were subjected to western blot analysis using anti-dMfn. There were two major bands (long and short forms) representing dMfn. The short form of dMfn is shown in Fig. 7D because the longer form was not detected in the *PINK1^−/−^* crosses. The dot indicates non-specific bands.
(TIF)

Figure S9 Phospho-polyUb seeds on the mitochondria generated by Parkin and PINK1 promote Parkin translocation. (**A**) Translocation efficiency of Myc-tagged Parkin WT in HeLa cells expressing Tom70^MTS^-4xUb WT or SA, which were treated with 10 µM valinomycin for 1 hr. The graph indicates means ±SEM of the percentages of cells exhibiting mitochondrial recruitment in three independent experiments, with ∼100 anti-Myc staining-positive cells counted per sample. * *p*<0.05 (two-tailed unpaired Student's *t*-test). Representative cell images are shown in (**B**, 3, 4 in the bottom). (**B**) A model of the chain reaction of Parkin translocation triggered by mitochondrial phospho-polyUb chains. 1, 2; Parkin C431S cannot generate polyUb chains itself, and its mitochondrial translocation depends on the properties of the Tom70^MTS^-4xUb chain (shown in blue). Tom70^MTS^-4xUb WT is subjected to PINK1 phosphorylation with valinomycin treatment. Thus, Tom70^MTS^-4xUb WT and SE have a similar ability to recruit Parkin. 3, 4; Parkin WT activated by Tom70^MTS^-4xUb WT or SE (shown in blue) can newly generate mitochondrial Ub chains (shown in green), which are phosphorylated by PINK1. The abundance of phospho-polyUb chains affects the efficiency of Parkin translocation. Thus, overexpression of Tom70^MTS^-4xUb WT or SE promotes Parkin translocation, whereas overexpression of Tom70^MTS^-4xUb SA diminishes Parkin translocation, as shown in (**A**). Representative cell images are also shown. Green, Parkin; red, Tom20 as a mitochondrial marker. Parkin signals are also shown as monochrome images.
(TIF)

Figure S10 The loss of HOIL-1 does not affect the mitochondrial translocation of Parkin. (**A**) *HOIL-1^+/+^* and *HOIL-1^−/−^* MEFs retrovirally introduced with GFP-Parkin were treated with 10 µM valinomycin for 4 hr. GFP-Parkin and the mitochondria were visualized with the GFP signal (green) and anti-Tom20 (red), respectively. The Parkin and Tom20 signals are also shown as monochrome images. Scale bar  = 20 µm. (**B**) Translocation efficiency of GFP-Parkin in *HOIL-1^+/+^* and *HOIL-1^−/−^* MEFs treated as in (**A**). The graph indicates means ±SEM of the percentages of cells exhibiting mitochondrial recruitment in three independent experiments, with ∼100 cells counted per sample. N.S., not significant by two-tailed unpaired Student's *t*-test.
(TIF)

Video S1 Tom70^MTS^ does not retain GFP-Parkin S65E at the mitochondria. FLIP imaging of HeLa cells transfected with GFP-Parkin S65E (green) along with Tom70^MTS^. Mitochondria were visualized with MitoTracker Red (red). Scale bar  = 5 µm. Related to Fig. 6A.
(MOV)

Video S2 Tom70^MTS^-4xUb SA does not retain GFP-Parkin S65E at the mitochondria. FLIP imaging of HeLa cells transfected with GFP-Parkin S65E (green) along with Tom70^MTS^-4xUb SA. Scale bar  = 5 µm. Related to Fig. 6A.
(MOV)

Video S3 Tom70^MTS^-4xUb SE retains GFP-Parkin S65E at the mitochondria. FLIP imaging of HeLa cells transfected with GFP-Parkin S65E (green) along with Tom70^MTS^-4xUb SE. Scale bar  = 5 µm. Related to Fig. 6A.
(MOV)
