# Supplementary figures and images for: Phosphorylation of Mitochondrial Polyubiquitin by PINK1 Promotes Parkin Mitochondrial Tethering
Source: PLoS Genet. 2014 Dec 4;10(12):e1004861. doi: 10.1371/journal.pgen.1004861 (PMC4256268; doi:10.1371/journal.pgen.1004861)

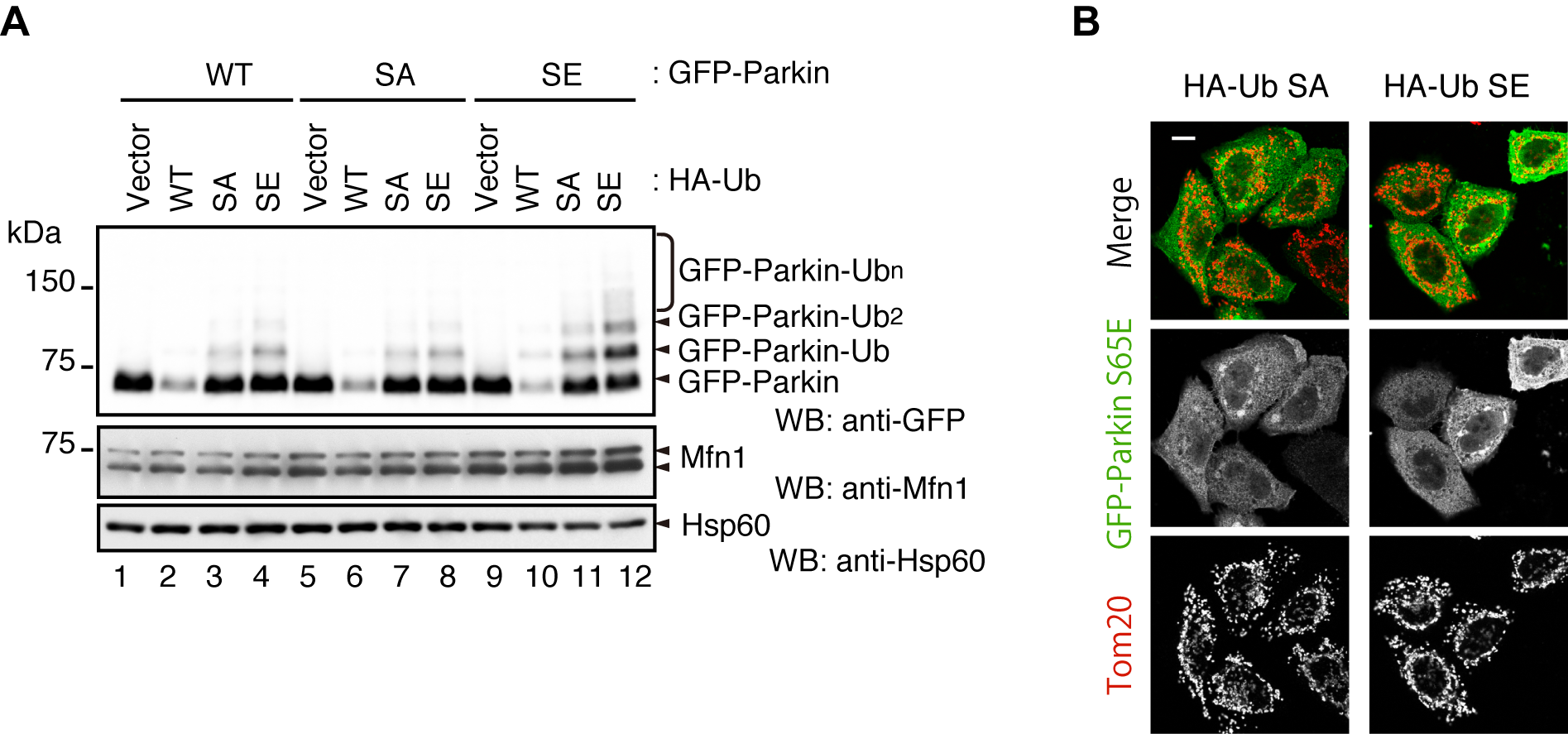

Supplement: Figure S1 — Phosphorylation of Ser65 in Ub and Parkin activate Parkin's E3 activity in an additive manner. (A) The autoubiquitination of GFP-Parkin is stimulated by Ub. Lysates from HEK293T cells transfected with the indicated combinations of cDNA were analysed by western blotting. The mitochondrial matrix protein Hsp60 served as a loading control. The expression of HA-Ub WT tended to reduce Parkin levels, suggesting that WT Ub is used for Parkin degradation rather than activation. (B) The coexpression of GFP-Parkin SE with HA-Ub SE does not stimulate the mitochondrial translocation of Parkin in HeLa cells. GFP-Parkin and mitochondria were visualized with GFP signal (green) and anti-Tom20 (red), respectively. Scale bar = 10 µm. (TIF) [file pgen.1004861.s001.tif]

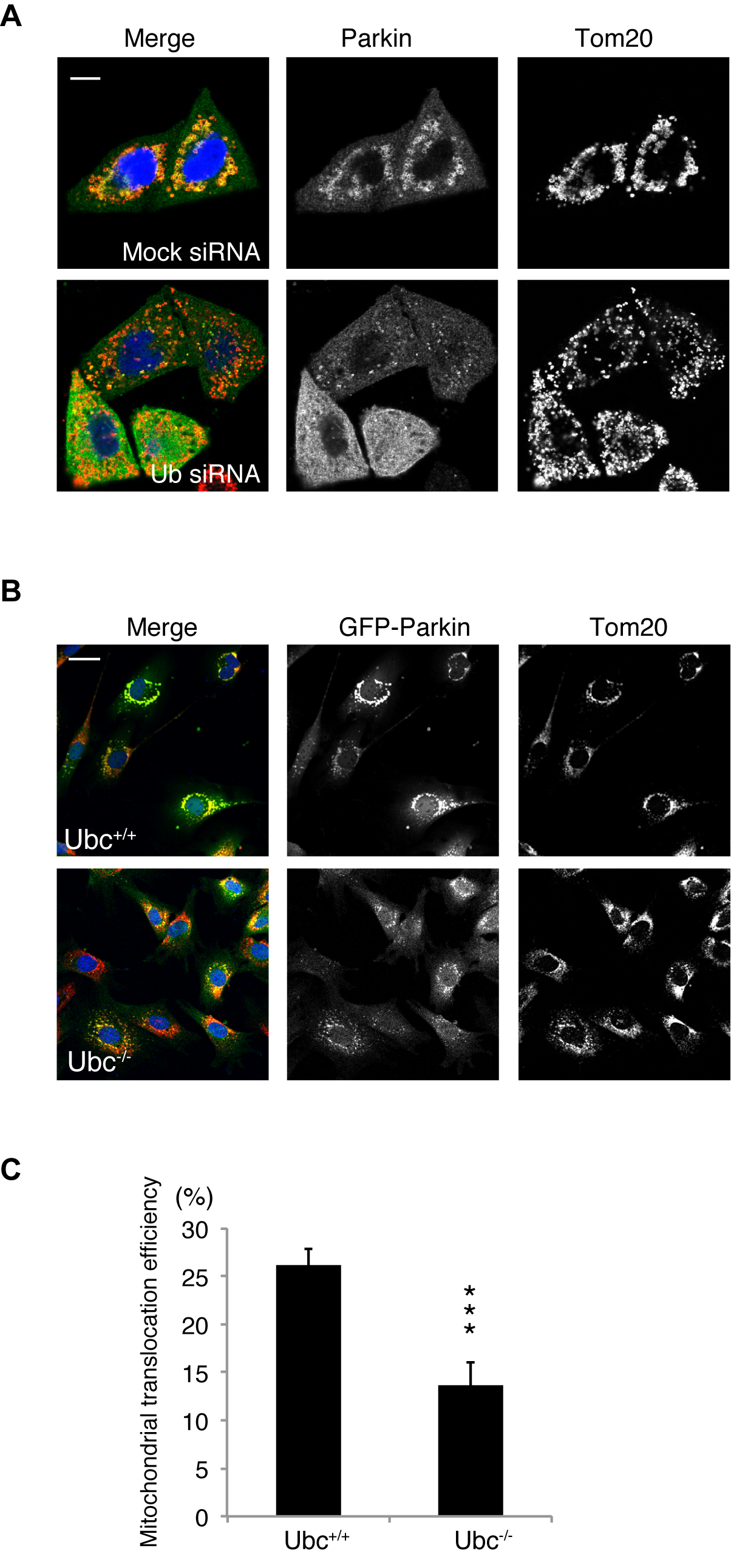

Supplement: Figure S2 — Reduction of Ub expression attenuates Parkin mitochondrial translocation. (A) Ub is required for Parkin translocation. HeLa cells stably expressing non-tagged Parkin were transfected with mock siRNA or an siRNA mixture for RPS27A, UBA52, UBB and UBC (2.5 nM each, Silencer, Life Technologies). At 48 hr post-transfection, the cells were treated with 10 µM CCCP for 30 min. Parkin and the mitochondria were visualized with anti-Parkin (green) and anti-Tom20 (red), respectively. The Parkin and Tom20 signals are also shown as monochrome images. (B, C) The reduced expression of Ub delays Parkin translocation to the depolarized mitochondria. (B) Ubc+/+ or Ubc−/− MEFs retrovirally introduced with GFP-Parkin were treated with 30 µM CCCP for 4.5 hr. GFP-Parkin and the mitochondria were visualized with the GFP signal (green) and anti-Tom20 (red), respectively. The Parkin and Tom20 signals are also shown as monochrome images. (C) The GFP signals colocalized with anti-Tom20 as in (B) were extracted using ImageJ. The mitochondrial translocation efficiency is presented by the percentage of cells with the mitochondrial GFP signals over 2-fold median fluorescence intensity in each image. The graph shows the means ±SEM in three independent experiments, with ∼90 cells counted per sample. *** p<0.001 (two-tailed unpaired Student's t-test). Scale bars = 10 µm in (A), 30 µm in (B). (TIF) [file pgen.1004861.s002.tif]

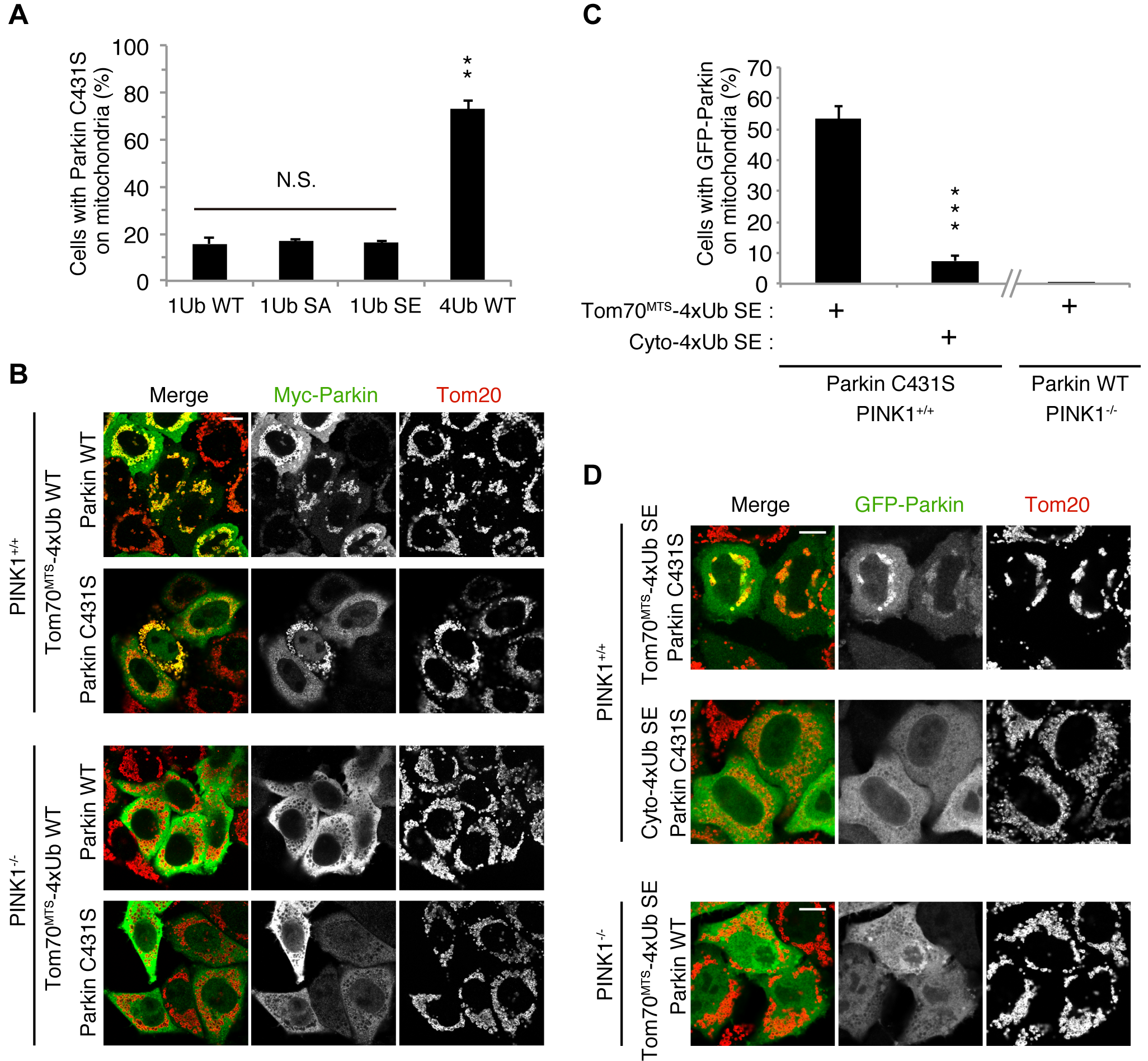

Supplement: Figure S3 — Neither mitochondrial monoUb nor cytosolic polyUb promotes Parkin recruitment. (A) Mitochondrial phospho-monoUb does not recruit Parkin C431S efficiently. The graph indicates means ±SEM of the percentages of cells exhibiting mitochondrial recruitment in three independent experiments. 1xUb WT, SA or SE vs. 4Ub WT, **p<0.01; N.S., not significant by the Tukey-Kramer test. (B) PINK1 is required for Parkin C431S recruitment by Tom70MTS-4xUb. A similar experiment as in Fig. 2A was performed using normal HeLa cells (PINK1+/+) and PINK1-deficient HeLa cells (PINK1−/−). Parkin WT was also included as controls. Scale bar = 10 µm. (C, D) Cytosolic 4xUb SE does not recruit Parkin C431S efficiently and Tom70MTS-4xUb SE fails to recruit Parkin WT to mitochondria in the absence of PINK1. HeLa cells (PINK1+/+) expressing GFP-Parkin C431S along with Tom70MTS-4xUb SE or 4xUb SE without Tom70MTS (Cyto-4xUb SE) and PINK1-deficient HeLa cells (PINK1−/−) expressing GFP-Parkin WT along with Tom70MTS-4xUb SE were treated as shown in Fig. 2. (C) The graph indicates means ±SEM of the percentages of cells exhibiting mitochondrial recruitment in three independent experiments, with ∼100 GFP-positive cells counted per sample. Cyto-4xUb SE vs. Tom70MTS-4xUb SE, ***p<0.001 (two-tailed unpaired Student's t-test). (D) Representative images as in (C). Scale bar = 10 µm. (TIF) [file pgen.1004861.s003.tif]

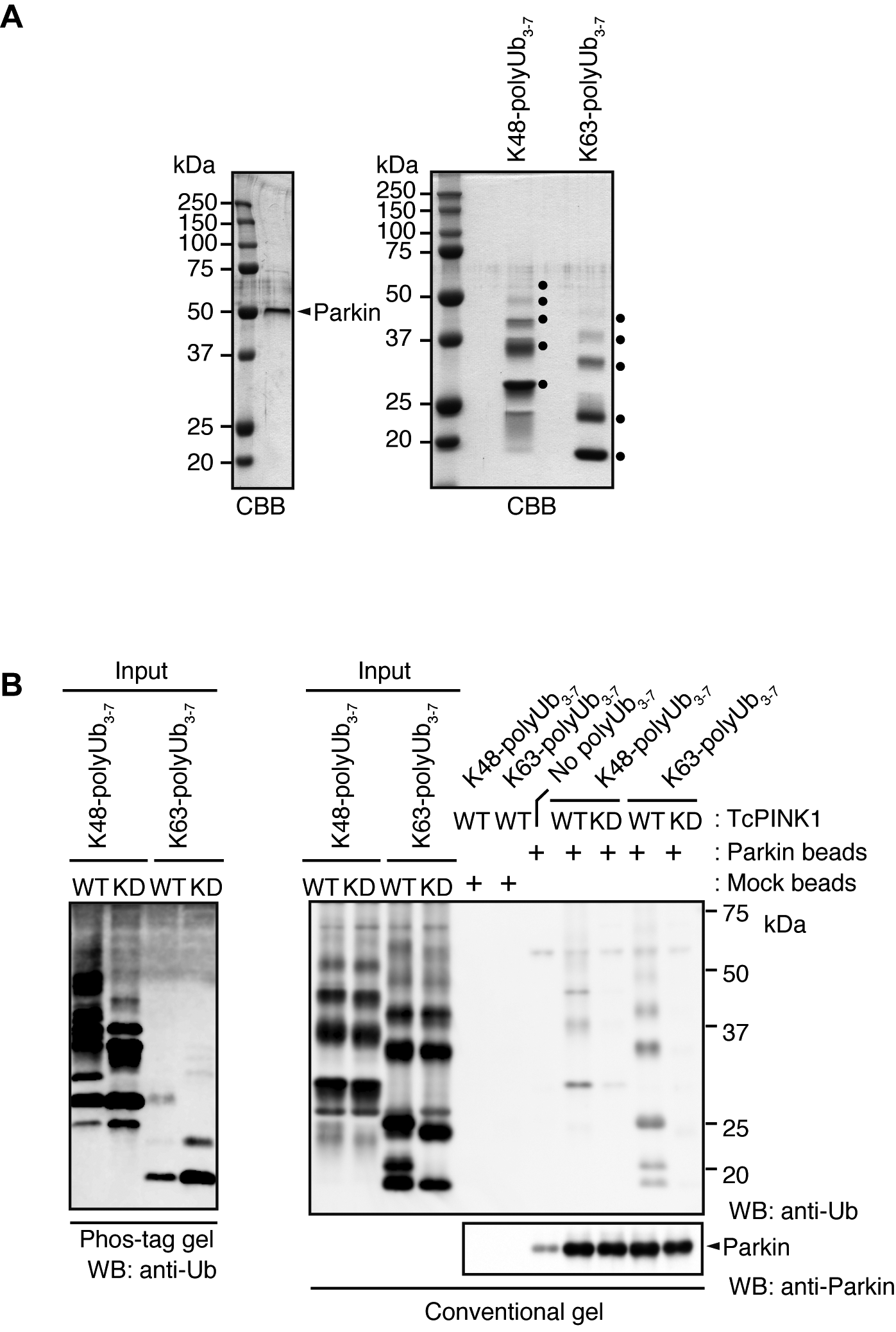

Supplement: Figure S4 — Parkin produced in bacteria binds to both K48-linked and K63-linked phospho-Ub chains. (A) Non-tag Parkin prepared from bacteria was visualized by CBB staining (left). K48-linked and K63-linked polyUb3–7 used in this study (right). (B) In vitro Parkin pull-down assay for linkage-specific phospho-Ub chains was performed as in Fig. 4. (TIF) [file pgen.1004861.s004.tif]

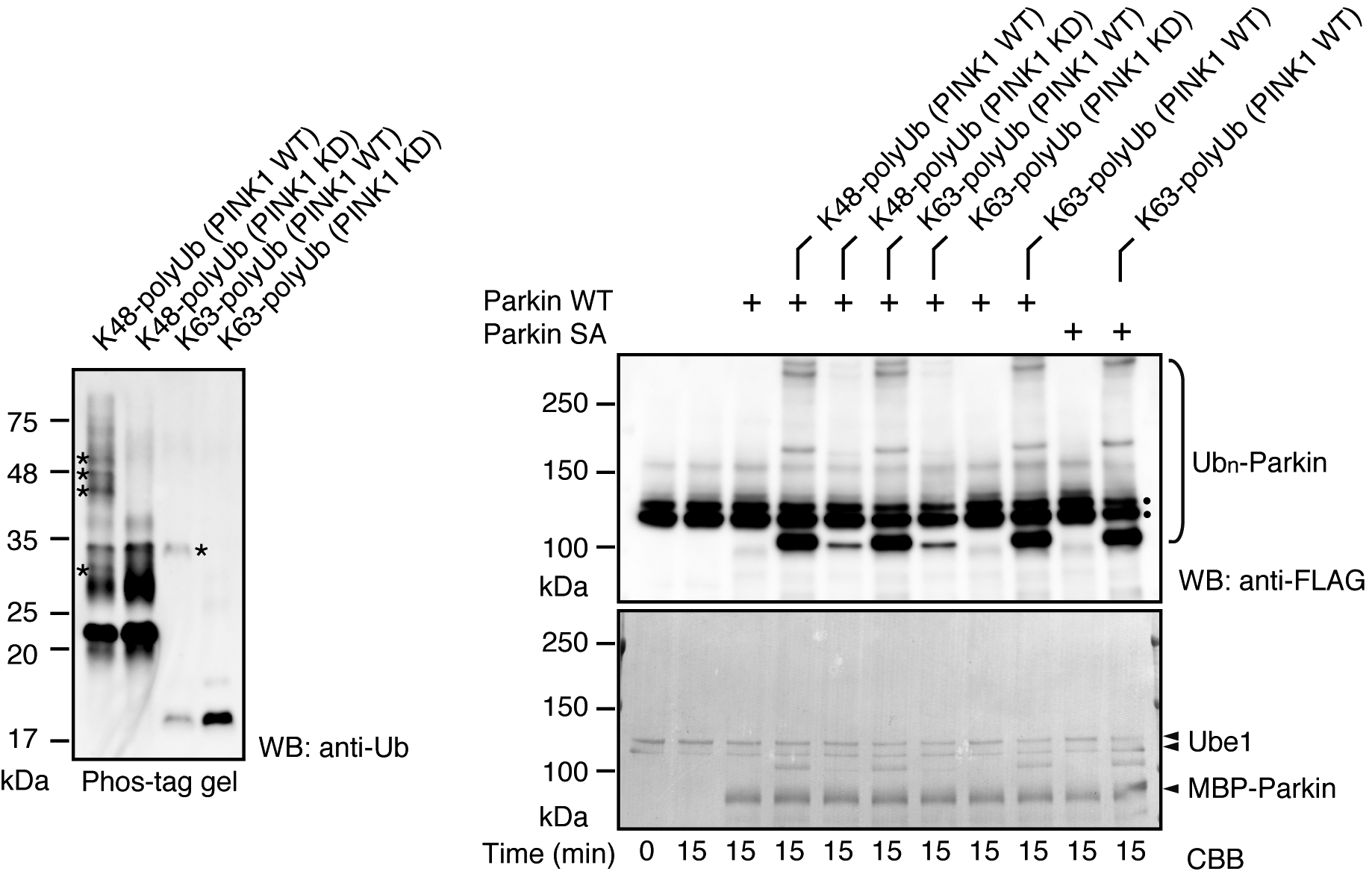

Supplement: Figure S5 — Parkin is activated by both K48-linked and K63-linked phospho-Ub chains. MBP-human Parkin WT and Ser65A (SA) were incubated for 15 min at 30°C with K48-polyUb3–7 or K63-polyUb3–7 pretreated with TcPINK1 WT or KD. Control reactions without MBP-Parkin, polyUb or incubation at 30°C were also performed through the same procedure. Dots indicate putative Ube1∼Ub bands. Phosphorylation of Ub chains (asterisks) was confirmed by Phos-tag western blot (Left). Note that Phos-tag polyacrylamide gel electrophoresis is sensitive to the conformation and charge of proteins, and does not always reflect their actual molecular mass. (TIF) [file pgen.1004861.s005.tif]

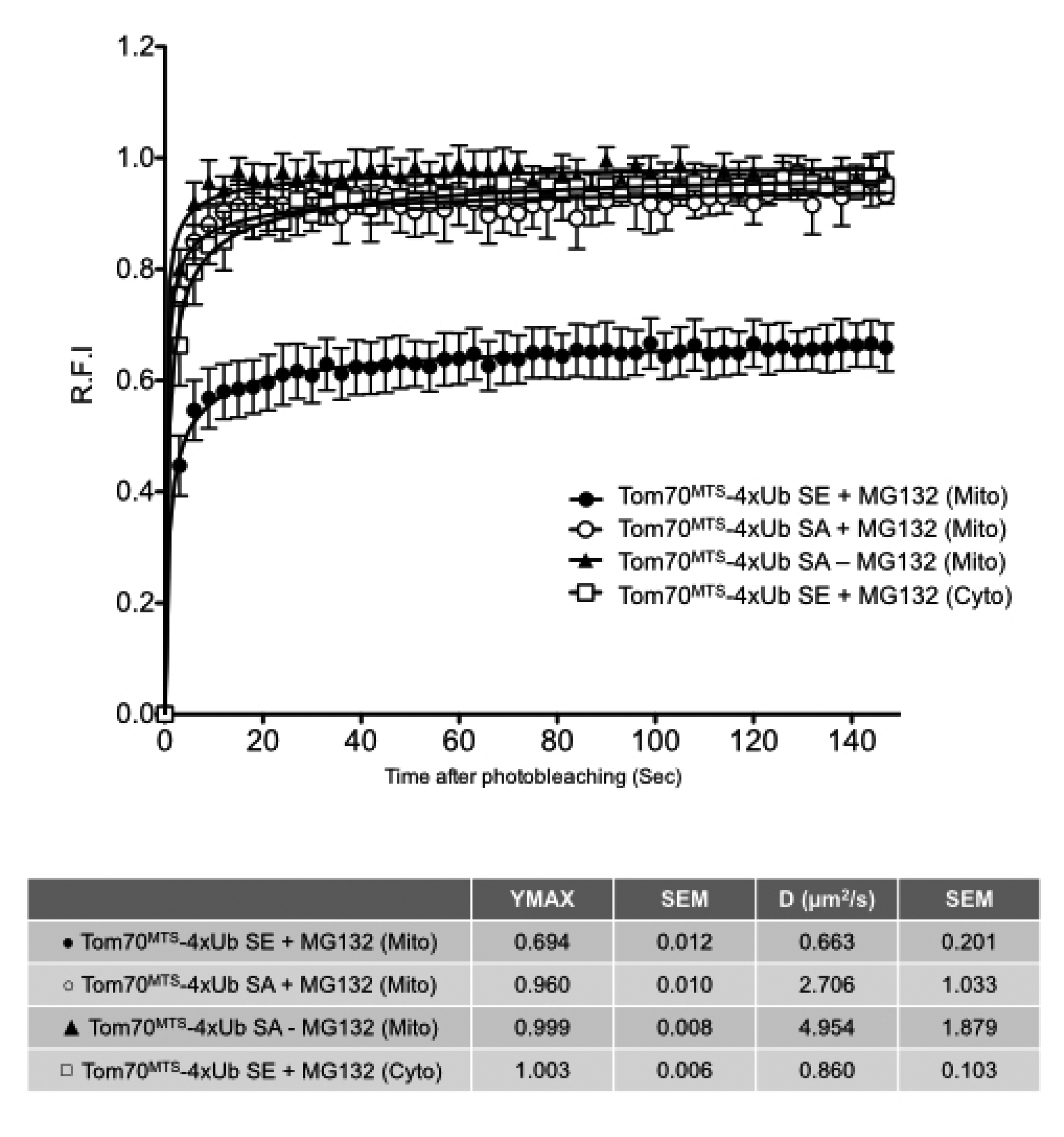

Supplement: Figure S6 — Mitochondrial phosphomimetic 4× Ub reduces the mobility of GFP-Parkin localized at the mitochondria. Quantitative FRAP analysis was performed as in Fig. 6 in the presence or absence of 2 µM MG132. RFI is represented as the mean ±SEM (n≥3). The values of D and YMAX indicate that Tom70MTS-4xUb SA does not recruit GFP-Parkin at the mitochondria (Mito) and MG132 treatment does not affect the diffusion of GFP-Parkin in the cytoplasm (Cyto). (TIF) [file pgen.1004861.s006.tif]

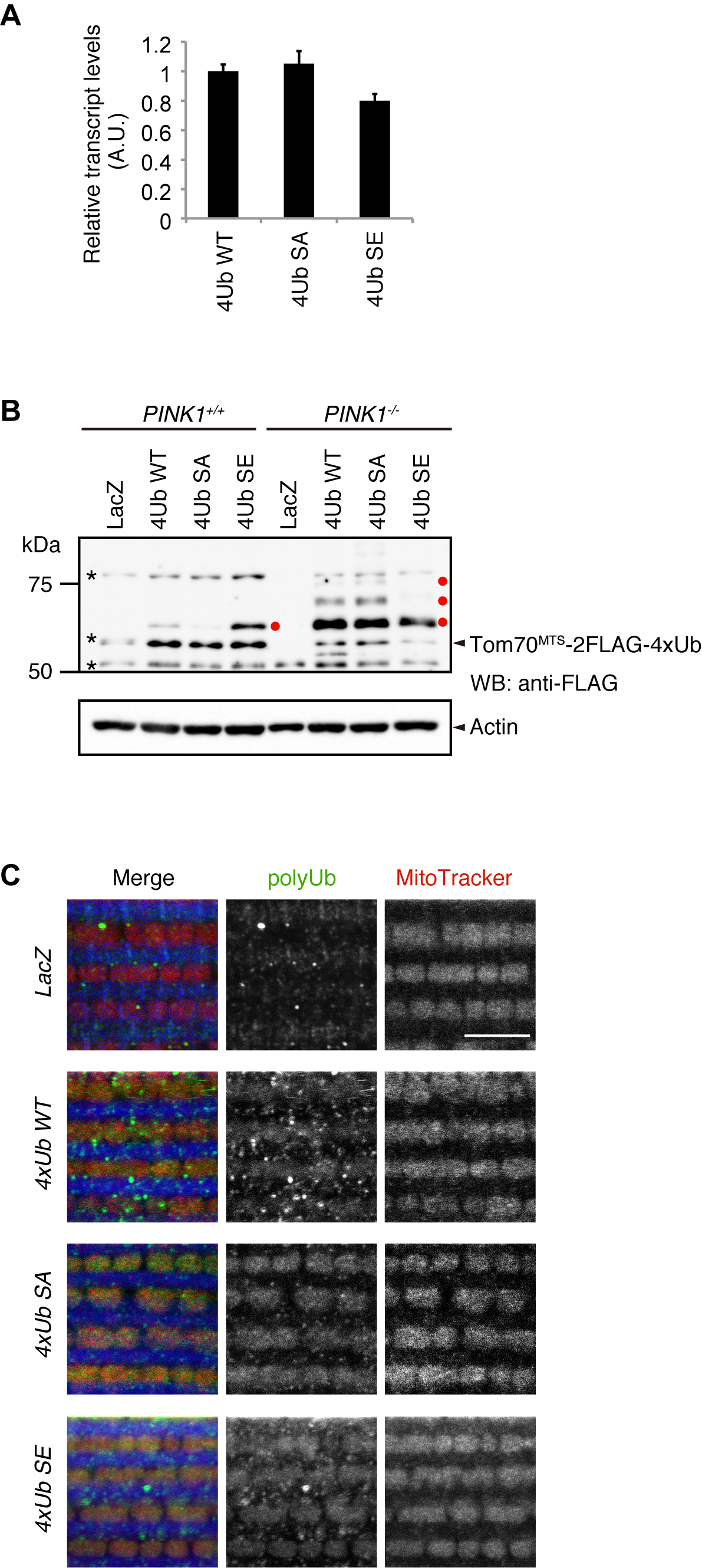

Supplement: Figure S7 — Expression of Tom70MTS-2FLAG-4Ub in the Drosophila thorax. (A) The levels of Tom70MTS-2FLAG-4Ub transcripts were measured using quantitative RT-PCR and were normalized by housekeeping rp49 levels. Total RNA was extracted from the thoraxes of 5-day-old adult male flies (n = 10). All measurements were performed in triplicate, and values represent the means ±SEM. Expression of 4Ub SE was reduced compared with 4Ub SA (p<0.01 by the Tukey-Kramer test) and there were no significant differences between other combinations. (B) The protein levels of Tom70MTS-2FLAG-4Ub in the thoraxes of 5-day-old adult male flies were examined using Western blot. Actin signal served as a loading control. Red dots and asterisks indicate putative endogenous Ub modifications and non-specific bands detected by the antibody. (C) Mitochondrial polyUb signals in Tom70MTS-2FLAG-4Ub-expressing flies. Fluorescent images of the indirect flight muscle in the indicated genotypes of 5- to 7-day-old adult flies are shown. LacZ expression was used as a control. PolyUb signals (green) were visualized with anti-polyUb antibody (FK2, 1∶100) and the muscle tissues were counterstained with MitoTracker (red) and phalloidin (blue). Scale bar = 5 µm. Expression of the transgenes was induced by the MHC-GAL4 driver in (A–C). (TIF) [file pgen.1004861.s007.tif]

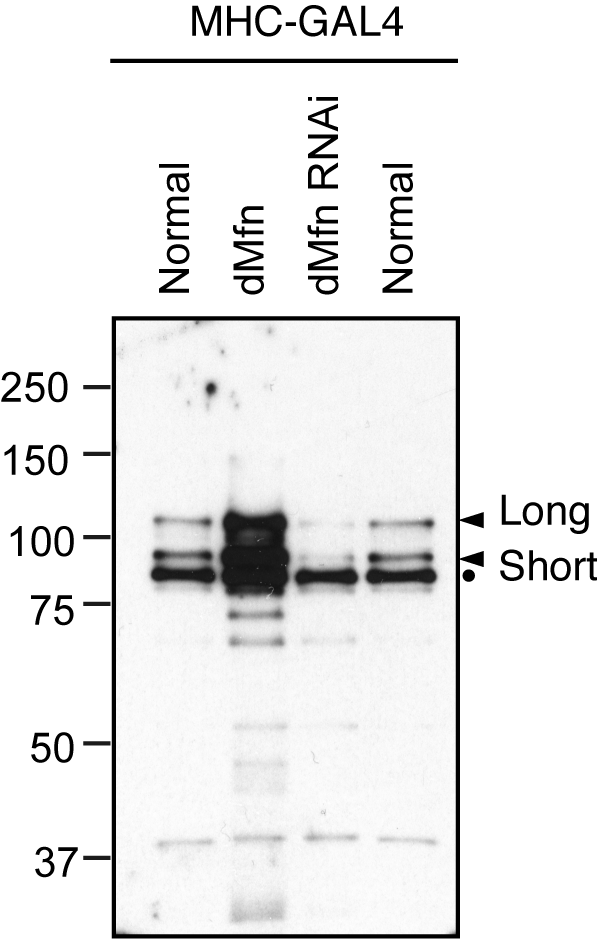

Supplement: Figure S8 — Specificity of anti-dMfn antibody. UAS-dMfn (a kind gift from Dr M. Feany) and UAS-dMfn RNAi (VDRC stock) were driven by MHC-GAL4. MHC-GAL4 crossed with w- was used as a normal control (Normal). Muscle tissues were subjected to western blot analysis using anti-dMfn. There were two major bands (long and short forms) representing dMfn. The short form of dMfn is shown in Fig. 7D because the longer form was not detected in the PINK1−/− crosses. The dot indicates non-specific bands. (TIF) [file pgen.1004861.s008.tif]

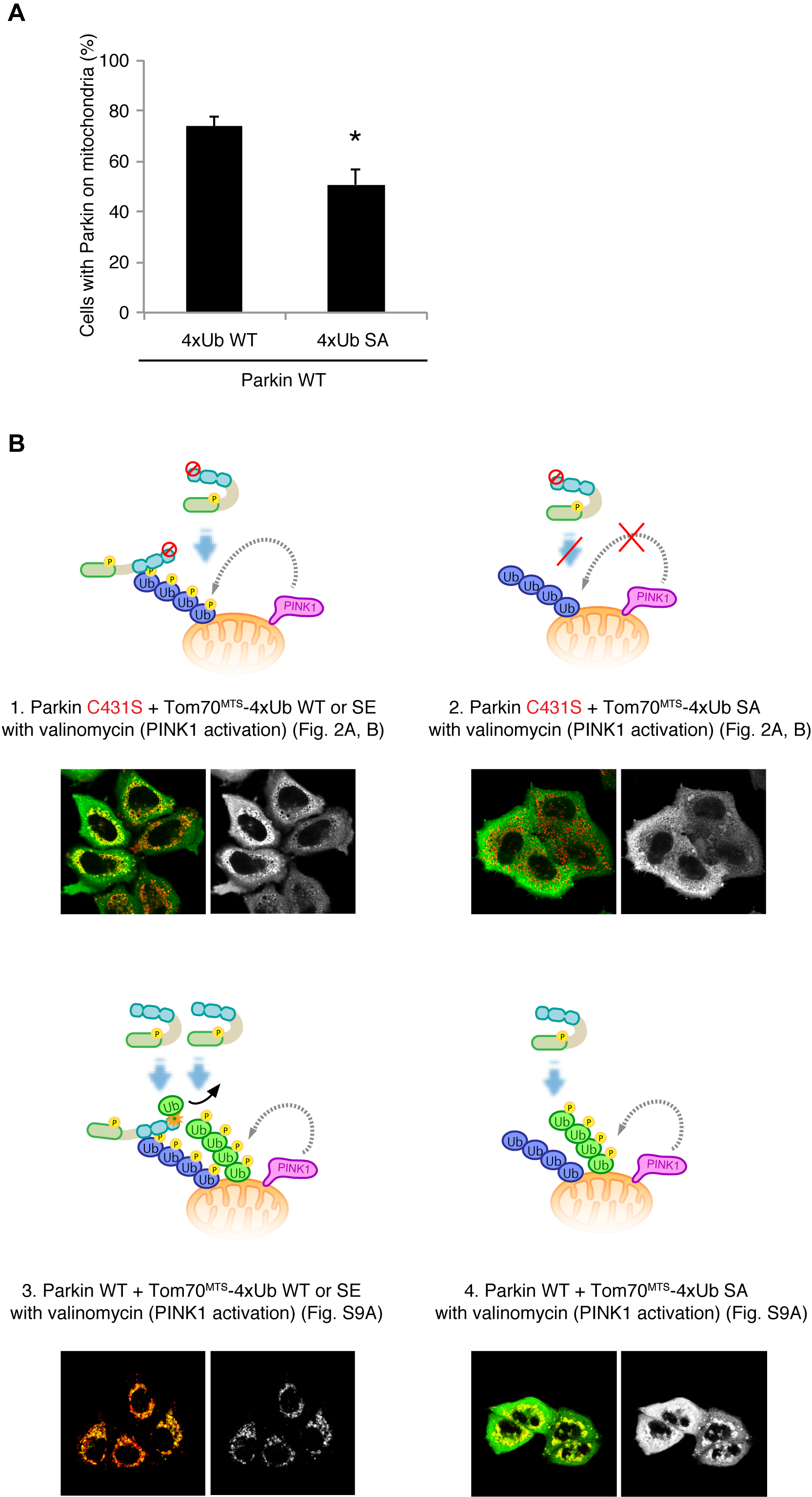

Supplement: Figure S9 — Phospho-polyUb seeds on the mitochondria generated by Parkin and PINK1 promote Parkin translocation. (A) Translocation efficiency of Myc-tagged Parkin WT in HeLa cells expressing Tom70MTS-4xUb WT or SA, which were treated with 10 µM valinomycin for 1 hr. The graph indicates means ±SEM of the percentages of cells exhibiting mitochondrial recruitment in three independent experiments, with ∼100 anti-Myc staining-positive cells counted per sample. * p<0.05 (two-tailed unpaired Student's t-test). Representative cell images are shown in (B, 3, 4 in the bottom). (B) A model of the chain reaction of Parkin translocation triggered by mitochondrial phospho-polyUb chains. 1, 2; Parkin C431S cannot generate polyUb chains itself, and its mitochondrial translocation depends on the properties of the Tom70MTS-4xUb chain (shown in blue). Tom70MTS-4xUb WT is subjected to PINK1 phosphorylation with valinomycin treatment. Thus, Tom70MTS-4xUb WT and SE have a similar ability to recruit Parkin. 3, 4; Parkin WT activated by Tom70MTS-4xUb WT or SE (shown in blue) can newly generate mitochondrial Ub chains (shown in green), which are phosphorylated by PINK1. The abundance of phospho-polyUb chains affects the efficiency of Parkin translocation. Thus, overexpression of Tom70MTS-4xUb WT or SE promotes Parkin translocation, whereas overexpression of Tom70MTS-4xUb SA diminishes Parkin translocation, as shown in (A). Representative cell images are also shown. Green, Parkin; red, Tom20 as a mitochondrial marker. Parkin signals are also shown as monochrome images. (TIF) [file pgen.1004861.s009.tif]

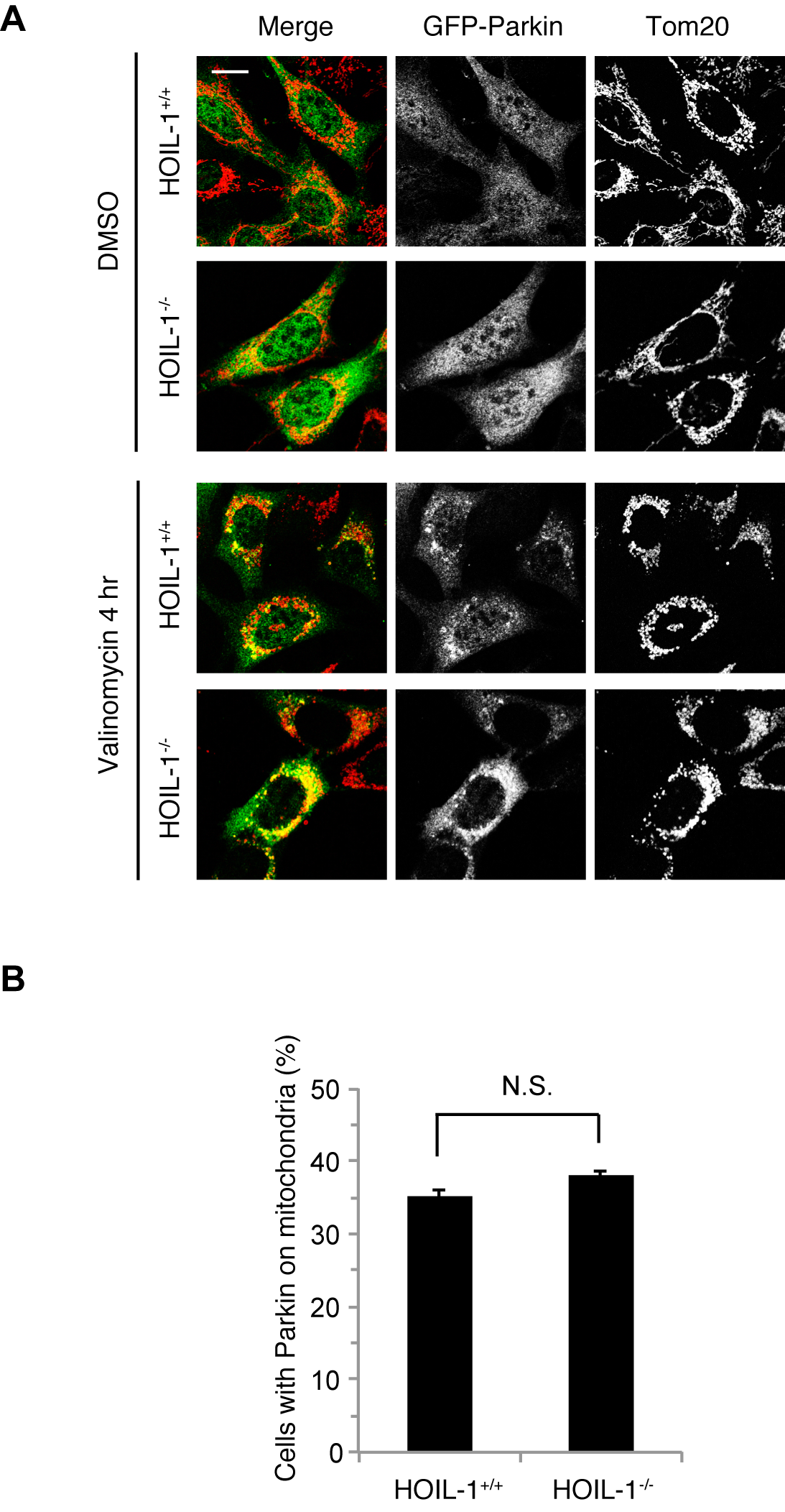

Supplement: Figure S10 — The loss of HOIL-1 does not affect the mitochondrial translocation of Parkin. (A) HOIL-1+/+ and HOIL-1−/− MEFs retrovirally introduced with GFP-Parkin were treated with 10 µM valinomycin for 4 hr. GFP-Parkin and the mitochondria were visualized with the GFP signal (green) and anti-Tom20 (red), respectively. The Parkin and Tom20 signals are also shown as monochrome images. Scale bar = 20 µm. (B) Translocation efficiency of GFP-Parkin in HOIL-1+/+ and HOIL-1−/− MEFs treated as in (A). The graph indicates means ±SEM of the percentages of cells exhibiting mitochondrial recruitment in three independent experiments, with ∼100 cells counted per sample. N.S., not significant by two-tailed unpaired Student's t-test. (TIF) [file pgen.1004861.s010.tif]
